# The Halogen Bonding Proclivity of the sp^3^ Sulfur Atom as a Halogen Bond Acceptor in Cocrystals of Tetrahydro-4*H*-thiopyran-4-one and Its Derivatives

**DOI:** 10.1021/acs.cgd.2c00793

**Published:** 2022-09-13

**Authors:** Vinko Nemec, Dominik Cinčić

**Affiliations:** Department of Chemistry, Faculty of Science, University of Zagreb, Horvatovac 102a, 10000 Zagreb, Croatia

## Abstract

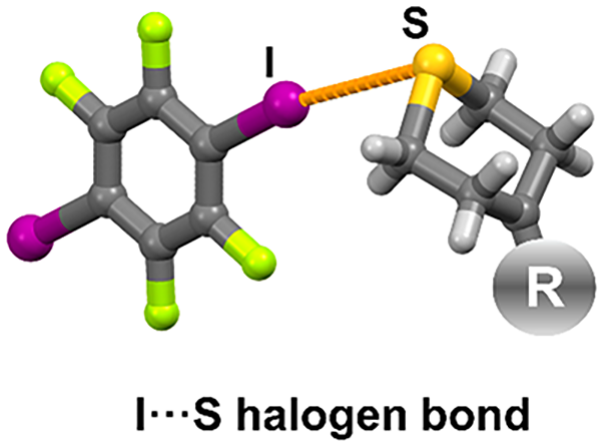

In this work, we present a systematic study of the capability of the sp^3^ hybridized sulfur atom for halogen bonding both in a small building block, tetrahydro-4*H*-thiopyran-4-one, and two larger ones derived from it, Schiff bases with a morpholine fragment on the other end of the molecule. These three building blocks were cocrystallized with six perhalogenated aromates: 1,4-diiodotetrafluorobenzene, 1,3,5-triiodotrifluorobenzene, 1,3-diiodotetrafluorobenzene, 1,2-diiodotetrafluorobenzene, iodopentafluorobenzene, and 1,4-dibromotetrafluorobenzene. Out of the 18 combinations, only 7 (39%) yielded cocrystals, although with a high occurrence of the targeted I···S halogen bonding motif in all cocrystals (71%), and in imine cocrystals the I···O_morpholine_ motif (100%) as well as, surprisingly, the I···N_imine_ motif (100%). The I···S halogen bonds presented in this work feature lower relative shortening values than those for other types of sulfur atoms; however, the sp^3^ sulfur atom could potentially be more specific an acceptor for halogen bonding.

## Abstract 2

Three building blocks, tetrahydro-4*H*-thiopyran-4-one and two Schiff bases derived from it and either 4-aminomorpholine or 4-(2-aminoethyl)morpholine, were cocrystallized with a series of six perhalogenated benzenes as halogen bond donors. Seven cocrystals were obtained, allowing a systematic study of I···S halogen bonding involving sp^3^ hybridized sulfur atoms.

In the field of crystal engineering of multicomponent crystals by using halogen bonds,^1^ sulfur is significantly less studied as an acceptor species relative to the various functional groups containing oxygen,^2−21^ nitrogen,^22−33^ or halogen atoms.^34−38^ In most systematic studies, sulfur as a halogen bond acceptor is present in the form of either the thiocyanate free ion or ligand,^39−42^ thiocarbonyl,^43−46^ or thioamide functional groups.^47,48^ Recent studies have also shown the halogen bond acceptor proclivity of the sp^3^ hybridized sulfur atom in dimethyl sulfide,^49^ 1-(4-pyridyl)-4-thiopyridine,^50^ dithiocarbamate metal complexes,^51^ and in phosphorothioate in a model biochemical system.^52^ Another interesting approach in the past two decades has been the utilization of the sulfur atom in 1,4-dithiane, 1,4-thioxane and thiomorpholine as a halogen bond acceptor, whether in binary cocrystals^53^ or metal complex adduct cocrystals.^54^ However, the limitation in that approach is the utilization of relatively small building blocks that cannot easily be expanded, and that as monodentate ligands they are relatively labile, meaning that they not as strongly bound to the metal center and can as a result be eliminated or easily exchanged during crystallization for other ligands.^55^ An ideal ligand in our case would be one that has been proven to be a reliable halogen bond acceptor, is sufficiently strongly bound to the metal center in a predictable manner, and is not hindered as an acceptor species after binding. A strategy that we have decided to pursue is the utilization of imines with one or several potential halogen bond acceptor species that are located either near the imine C=N bond^16,17,56^ or slightly spaced out from it and on a flexible “arm”.

To that end, in this work, we have selected tetrahydro-4*H*-thiopyran-4-one (**tpyr**), a small building block with two potentially competing halogen bond acceptors, the cyclic sulfur and carbonyl oxygen atoms. This building block was then expanded by solution syntheses with 4-aminomorpholine (**am**) or 4-(2-aminoethyl)morpholine (**aem**) into imines **tpyram** and **tpyraem**, respectively. The two imines contain three primary competing halogen bond acceptors, the cyclic sulfur, morpholinyl nitrogen and morpholinyl oxygen atoms. Although the imine nitrogen atom or the imine C=N bond, could theoretically function as a halogen bond acceptor, a Cambridge Structural Database survey shows that of 1110 structures containing C–C=N–C and C–X structural motifs, where X = I, Br, and N is an acyclic nitrogen atom, only 22 (2.0%) feature either C–X···C_imine_ or C–X···N_imine_ halogen bonds.^57^ The three building blocks, **tpyr**, **tpyram** and **tpyraem**, were then cocrystallized with a series of perhalogenated benzenes, 1,4-diiodotetrafluorobenzene (**14tfib**), 1,3-diiodotetrafluorobenzene (**13tfib**), 1,2-diiodotetrafluorobenzene (**12tfib**), iodopentafluorobenzene (**ipfb**), 1,3,5-triiodotrifluorobenzene (**135tfib**), and 1,4-dibromotetrafluorobenzene (**14tfbb**) (Scheme 1).

**Scheme 1:**
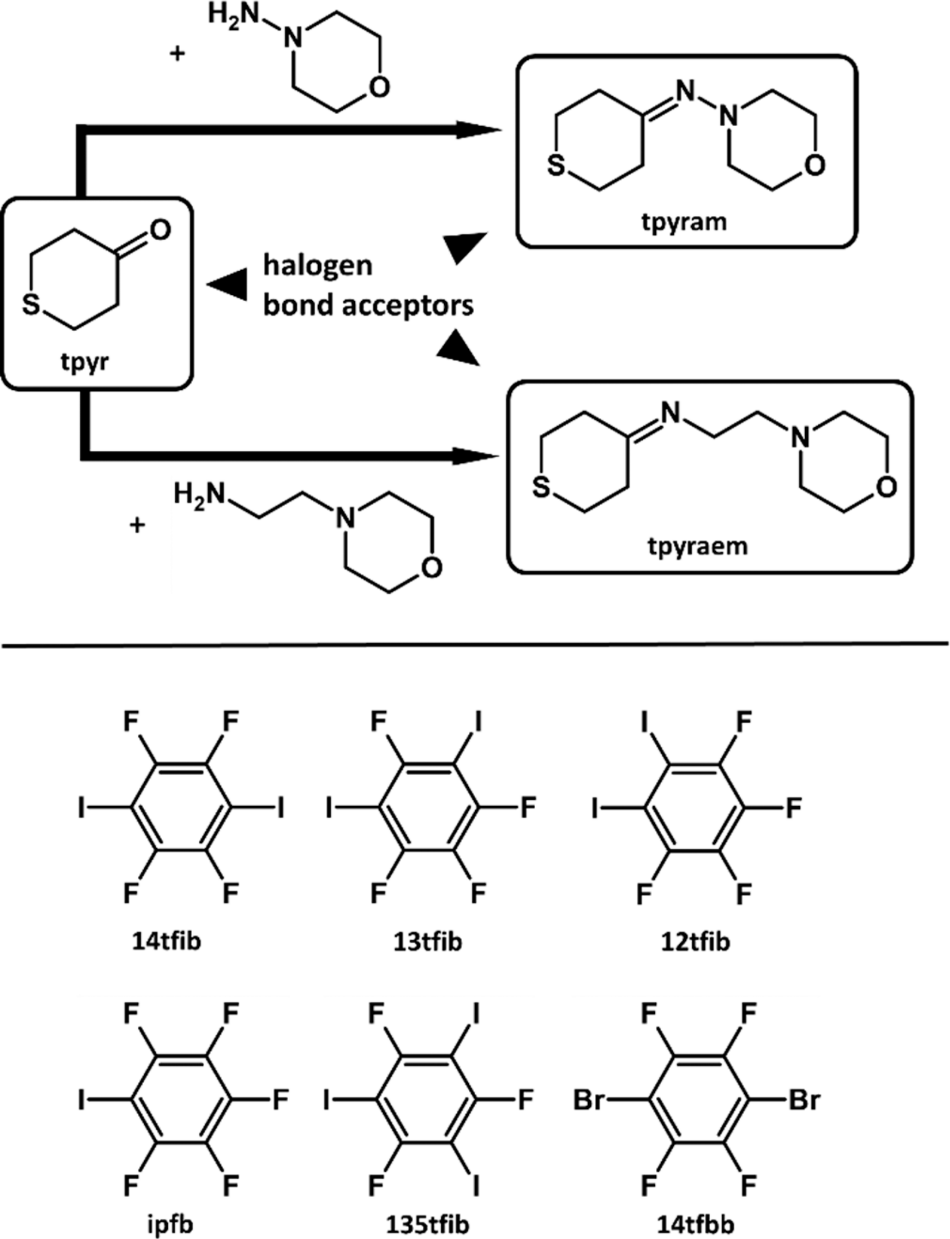
Molecular Structures of Halogen Bond Acceptors **tpyr**, **tpyram**, and **tpyraem** and the Halogen Bond Donors Used in This Study

Cocrystallization experiments for the solid halogen bond acceptors, **tpyr** and **tpyram**, were performed mechanochemically and by crystallization from solutions in order to obtain bulk products and single crystals. Since our **tpyraem** samples did not crystallize at room temperature, aliquots of a **tpyraem** methanol solution have been used in the cocrystallization experiments (see SI for details). Crystallization experiments were performed by dissolving a reactant mixture in an appropriate solvent, with or without heating, followed by letting the solvent or solvent mixture cool down and evaporate at room temperature. Mechanochemical experiments were performed on a Retsch MM200 Shaker Mill using stainless steel jars and stainless steel balls under normal laboratory conditions (temperature ca. 25 °C, 40–60% relative humidity, see SI for details).^58^ The obtained products were characterized by differential scanning calorimetry (DSC), powder (PXRD), and single-crystal X-ray diffraction (SCXRD).

A total of 7 new cocrystals have been synthesized and then characterized by the used analytical techniques. They are (**tpyr**)_2_(**14tfib**), (**tpyr**)(**135tfib**), (**tpyram**)_2_(**14tfib**)_3_, (**tpyram**)(**135tfib**), (**tpyram**)_2_(**14tfbb**)_3_, (**tpyraem**)(**14tfib**), and (**tpyraem**)_2_(**135tfib**)_5_. No new cocrystal products have been obtained at room temperature with either **ipfb**, **12tfib**, or **13tfib**. Halogen bonding to sulfur is present in 5 out of the 7 obtained cocrystals (71%). All 5 imine cocrystals feature halogen bonding with both the morpholine oxygen atom and, suprisingly, the imine nitrogen atom (Table 1 and Figure 1).

**Table 1:** Halogen Bond Lengths (d), Angles (∠), and Relative Shortenings (R.S.) of D···A Distances in the Herein Prepared Cocrystals^a^

cocrystal	D···A	acceptor moiety	*d* (D···A)/Å	∠ (C–D···A)/°	R.S.^b^/%
**(tpyr)_2_(14tfib)**	I1···S1	sp^3^ sulfur	3.384	172.7	10.5
**(tpyr)(135tfib)**	I1···O1	carbonyl	2.975	177.7	15.0
	I2···S1	sp^3^ sulfur	3.288	178.5	13.0
	I3···I2	donor iodine	3.791	166.9	4.3
**(tpyram)_2_(14tfib)_3_**	I2···O1	morpholine	3.097	171.1	11.5
	I3···S1	sp^3^ sulfur	3.556	169.9	5.9
	I1···N1	imine	3.097	175.9	12.3
**(tpyram)_2_(14tfbb)_3_**	Br2···O1	morpholine	3.043	172.6	9.7
	Br1···S1	sp^3^ sulfur	3.412	168.2	6.5
	Br3···N1	imine	3.079	174.5	9.4
**(tpyram)(135tfib)**	I1···O1	morpholine	2.927	172.5	16.3
	I4···O2	morpholine	2.922	175.3	16.5
	I7···O3	morpholine	2.892	175.9	17.4
	I10···O4	morpholine	2.923	172.0	16.5
	I3···N1	imine	3.105	168.5	12.0
	I5···N3	imine	3.153	173.6	10.7
	I9···N5	imine	3.107	173.4	12.0
	I11···N7	imine	3.089	169.8	12.5
**(tpyraem)(14tfib)**	I1···N2	morpholine	2.936	167.1	16.8
	I2···N1	imine	2.849	172.8	19.3
**(tpyraem)(135tfib)**	I6···S1	sp^3^ sulfur	3.452	171.2	8.7
	I4···O1	morpholine	2.943	167.6	15.9
	I1···N2	morpholine	3.034	173.3	14.1
	I2···N1	imine	3.107	170.4	12.0
	I8···I6	donor iodine	3.947	164.5	0.3
	I7···I4	donor iodine	3.790	159.3	4.3

a *T* = 295 K.

b Calculated according to the formula: , according to Bondi’s van der Waals radii^59^

**Figure 1:**
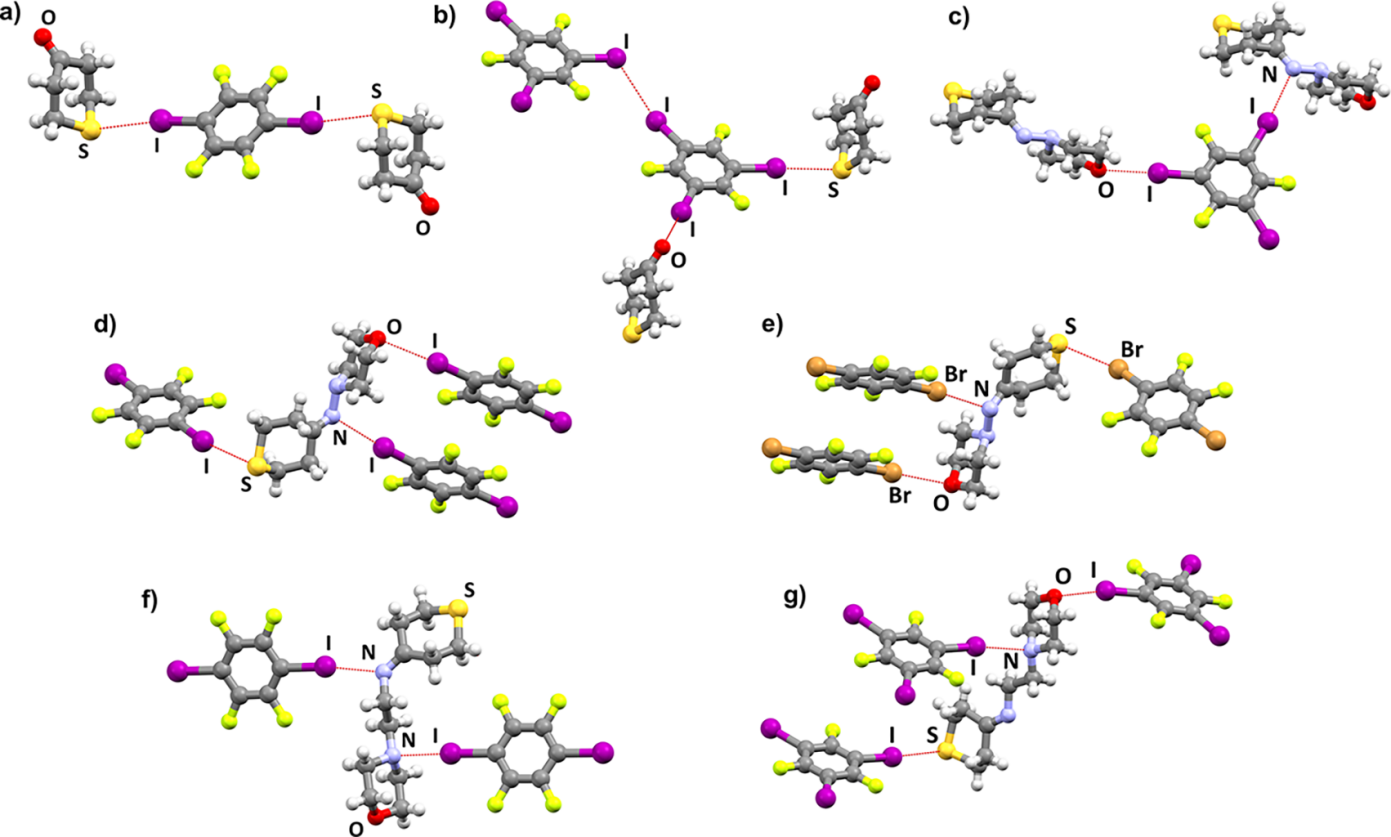
Parts of crystal structures in (a) (**tpyr**)_2_(**14tfib**), (b) (**tpyr**)(**135tfib**), (c) (**tpyram**)(**135tfib**), (d) (**tpyram**)_2_(**14tfib**)_3_, (e) (**tpyram**)_2_(**14tfbb**)_3_, (f) (**tpyraem**)(**14tfib**), (g) (**tpyraem**)_2_(**135tfib**)_5_.

Regarding the two cocrystals of the smaller halogen bond acceptor, **tpyr**, it is noticeable that the different donor atom positioning leads to the formation of significantly differently supramolecular motifs. In the cocrystal with the linear donor **14tfib**, **tpyr** molecules form 2D networks through C–H···O hydrogen bonding, and these networks are connected by I···S halogen bonding into a 3D network. On the other hand, in the cocrystal with the potentially tritopic^60^ and nonlinear donor **135tfib**, only halogen bonds are formed with the oxygen and sulfur atoms, leading to the formation of first, a halogen bonded chain, and then a halogen bonded layer when combined with I···I halogen bonds between donor molecules themselves. The only significant intermolecular hydrogen bonds are of C–H···F type, and they connect two adjacent halogen bonded layers. The lack of cocrystals with all other halogen bond donors is most likely a combination of the effects of inadequate donor atom positioning and donor atom strength when compared with the strength of homomeric interactions in pure **tpyr**. Pure **tpyr** features a very closely packed structure with six C–H···O hydrogen bonds per **tpyr** molecule (refcode OWEKEH).^61^ The crystal structures of both polymorphs of pure **tpyram** (this work, see SI) on the other hand feature fewer C–H···O hydrogen bonds which connect the molecules into chains (form I, two hydrogen bonds per molecule) or layers (form II, four hydrogen bonds per molecule). This presumption would explain why **14tfbb**, as a geometrically equivalent donor to **14tfib**, but participating in usually weaker intermolecular contacts, does not form a cocrystal with **tpyr** but does with **tpyram**, as well as why stronger but geometrically less adequate donors **12tfib** and **13tfib** do not form cocrystals with either **tpyr**, **tpyram** or **tpyraem**. Cocrystals of **tpyram** with linear donors **14tfib** and **14tfbb** are isomorphous, with **tpyram** molecules alternatingly bridged by one donor molecule forming X···S halogen bonds and then two donor molecules forming X···N_imine_ and X···O halogen bonds, leading to the formation of supramolecular chains in the crystal structures (X = I, Br). Additionally, only C–H···F hydrogen bonds are present in these two structures. In the **tpyram** cocrystal with **135tfib**, each **135tfib** molecule is a ditopic halogen bond donor, forming I···N_imine_ and I···O halogen bonds. The third iodine atom is oriented toward the area of negative electrostatic potential around a fluorine atom and the sigma-hole region of an iodine atom participating in the aforementioned I···O halogen bond. This combination of motifs leads to the formation of chains that are then connected into a 2D network by C–H···F contacts in two of the symmetrically inequivalent layers, or by a combination of C–H···O hydrogen bonds, C–H···I and C–H···F contacts in the other two layers. Layers are interconnected by a combination of out-of-plane C–H···π_135tfib_ and C–H···F contacts. It is worth noting that the sulfur atom participates in neither hydrogen nor halogen bonding in any of the layers. Cocrystals with the more flexible **tpyraem** molecule allow for halogen bonding with both the imine and morpholinyl nitrogen atoms. In the cocrystal with the linear **14tfib** donor, this arrangement of I···N halogen bonds leads to the formation of halogen bonded chains which are then connected into a 2D network by C–H···O hydrogen bonds and further into a 3D network by C–H···I contacts. The sulfur atom again does not participate in hydrogen or halogen bonding. On the other hand, the cocrystal with **135tfib** features halogen bonds with every potential halogen bond acceptor species. Alongside the I···N halogen bonds, both I···O and I···S halogen bonds are present, as well as some C–H···I and C–H···F contacts. Overall, the structure is easiest to describe as layers of **135tfib** molecules which are bridged by interspersed **tpyraem** molecules.

Thermal analysis of the reactants and cocrystals obtained in this work (data summarized in Table 2, see SI for DSC curves) shows one easily identifiable trend, and that is that the cocrystal melting or decomposition temperatures are connected to the melting points of donors used. Cocrystals with **14tfib** have lower onset temperatures than **135tfib** cocrystals with the same acceptor, and the one **14tfbb** cocrystal has the lowest onset temperature in its cocrystal series. The previously observed trend that cocrystal melting and decomposition temperatures tend to be located between the melting points of coformers^62,63^ mostly holds as well, although there are two exceptions. The onset temperature for the (**tpyram**)(**14tfbb**) cocrystal is below the melting points of both coformers, while the onset temperature for the (**tpyraem**)(**14tfib**) cocrystal is very similar to the melting point of pure **14tfib**, probably corresponding to simultaneous decomposition of the cocrystal and melting of the donor.

**Table 2:** Reactant and Cocrystal Melting or Decomposition Onset Temperatures Determined by the DSC Method for the Reactants and Products

compound	onset temperature/°C
**14tfib**	106.4
**14tfbb**	79.4
**135tfib**	152.8
**tpyr**	61.6
**tpyram** form I	82.3
**tpyram** form II	83.0
**(tpyr)_2_(14tfib)**	62.7
**(tpyr)(135tfib)**	127.6
**(tpyram)_2_(14tfib)_3_**	101.2
**(tpyram)_2_(14tfbb)_3_**	66.6
**(tpyram)(135tfib)**	117.9
**(tpyraem)(14tfib)**	107.0
**(tpyraem)(135tfib)**	119.0

To conclude, the sp^3^ sulfur atom in tetrahydro-4*H*-thipyran-4-one and its derivatives participated in halogen bonding in 5 out of 7 obtained cocrystals, with relative shortening (R.S.) values of 5.9–13.0%. These values fall well below the R.S. values of other acceptor species, with morpholinyl oxygen and imine nitrogen atoms being clear winners both in terms of relative shortening (11.5–17.5% for oxygen, 10.7–19.3% for nitrogen) and because they participate in halogen bonding in all applicable cocrystals. Simple comparisons with literature data for other types of acceptor sulfur atoms (thione, thiocyanate, or thioamide) show that these halogen bonds feature comparable or slightly higher upper limits on R.S. values (albeit at lower temperatures) to those in this work (6.0–17% for thiones,^43,45,46^ 7.6–17% for thioamides,^47,48^ and 5.8–16.4% for thiocyanate species^39−42,57^), as well as that the more exposed sulfur atoms or ions can participate in multiple halogen and hydrogen bonds. This apparent disadvantage of the hindered cyclic sp^3^ sulfur can also be considered an advantage, because it could have less propensity for hydrogen bonding. No hydrogen bonding with the sulfur atom has been observed in the obtained cocrystals, in pure **tpyram** (this work), nor in pure **tpyr**.^61^ Overall, while this work proves that there is potential in using cyclic sp^3^ sulfur atoms as halogen-bond-specific acceptors, more research is needed to ascertain their limitations and whether it is possible to improve their acceptor capabilities, for example by adding appropriate electron-donating groups near the sulfur atom. In their current form, these atoms seem to be more appropriate as secondary acceptor species, requiring greater consideration of the supramolecular connectivity and packing obtained through primary halogen and hydrogen bonding, neither of which is as yet a trivial challenge.

## Supporting Information Available

Experimental details, including DSC curves, PXRD patterns, and single crystal diffraction data (PDF)
