# Supplementary material for: The Halogen Bonding Proclivity of the sp3 Sulfur Atom as a Halogen Bond Acceptor in Cocrystals of Tetrahydro-4H-thiopyran-4-one and Its Derivatives
Source: Cryst Growth Des. 2022 Sep 13;22(10):5796–801. doi: 10.1021/acs.cgd.2c00793 (PMC9553023; doi:10.1021/acs.cgd.2c00793)
Supplement: Supplementary file 1 — cg2c00793_si_001.pdf [file cg2c00793_si_001.pdf]

SUPPORTING INFORMATION

The halogen bonding proclivity of the *sp*³ sulfur atom as a halogen bond acceptor in cocrystals of tetrahydro-4*H*-thiopyran-4-one and its derivatives

Vinko Nemec and Dominik Cinčić*

Department of Chemistry, Faculty of Science, University of Zagreb, Horvatovac 102a, HR-10000 Zagreb, Croatia

Email: dominik@chem.pmf.hr
Fax: +385 1 4606 341
Tel: +385 1 4606 362

Table of Contents

**Experimental details**	Mechanochemical syntheses, solution syntheses, thermal analysis, powder X-ray diffraction experiments, single crystal X-ray diffraction experiments	4–8
**Table S1.**	Mechanochemical synthesis parameters and results. The samples were milled for 20 minutes on a Retsch MM200 shaker mill set at 25 Hz vibration frequency, using Retsch 5 mL stainless steel jars and 1 stainless steel ball 7mm in diameter. **tpyraem** was used as a methanol solution, c = 1 mmol mL^{-1}.	6
**Table S2.**	Crystal data and refinement details for the prepared compounds.	9–11
**Figure S1.**	Molecular structure of (**tpyr**)$_2$(**14tfib**) showing the atom-labeling scheme. Displacement ellipsoids are drawn at the 50 % probability level, and H atoms are shown as small spheres of arbitrary radius.	12
**Figure S2.**	Molecular structure of (**tpyr**)(**135tfib**) showing the atom-labeling scheme. Displacement ellipsoids are drawn at the 50 % probability level, and H atoms are shown as small spheres of arbitrary radius.	12
**Figure S3.**	Molecular structure of (**tpyram**)$_2$(**14tfib**)$_3$ showing the atom-labeling scheme. Displacement ellipsoids are drawn at the 50 % probability level, and H atoms are shown as small spheres of arbitrary radius.	13
**Figure S4.**	Molecular structure of (**tpyram**)$_2$(**14tfbb**)$_3$ showing the atom-labeling scheme. Displacement ellipsoids are drawn at the 50 % probability level, and H atoms are shown as small spheres of arbitrary radius.	13
**Figure S5.**	Molecular structure of (**tpyram**)(**135tfib**) showing the atom-labeling scheme. Displacement ellipsoids are drawn at the 50 % probability level, and H atoms are shown as small spheres of arbitrary radius.	14
**Figure S6.**	Molecular structure of (**tpyraem**)(**14tfib**) showing the atom-labeling scheme. Displacement ellipsoids are drawn at the 50 % probability level, and H atoms are shown as small spheres of arbitrary radius.	14
**Figure S7.**	Molecular structure of (**tpyraem**)$_2$(**135tfib**)$_5$ showing the atom-labeling scheme. Displacement ellipsoids are drawn at the 50 % probability level, and H atoms are shown as small spheres of arbitrary radius.	15
**Figure S8.**	Molecular structure of **tpyram** form I, showing the atom-labeling scheme.	15

Displacement ellipsoids are drawn at the 50 % probability level, and H atoms are shown as small spheres of arbitrary radius.

Figure S9. Molecular structure of **tpyram** form II, showing the atom-labeling scheme. Displacement ellipsoids are drawn at the 50 % probability level, and H atoms are shown as small spheres of arbitrary radius. 16

Figure S10. PXRD patterns: a) calculated pattern from (**tpyr**)$_2$(**14tfib**) single crystal data, b) bulk product obtained by grinding **tpyr** and **14tfib** in a 2:1 stoichiometric ratio in the presence of 10.0 μL of nitromethane. 17

Figure S11. PXRD patterns: a) calculated pattern from (**tpyr**)(**135tfib**) single crystal data, b) bulk product obtained by grinding **tpyr** and **135tfib** in a 1:1 stoichiometric ratio in the presence of 10.0 μL of nitromethane. 17

Figure S12. PXRD patterns: a) calculated pattern from (**tpyram**)$_2$(**14tfib**)$_3$ single crystal data, b) product obtained by grinding **tpyram** and **14tfib** in a 2:3 stoichiometric ratio in the presence of 10.0 μL of nitromethane. 18

Figure S13. PXRD patterns: a) calculated pattern from (**tpyram**)$_2$(**14tfbb**)$_3$ single crystal data, b) product obtained by grinding **tpyram** and **14tfbb** in a 2:3 stoichiometric ratio in the presence of 10.0 μL of nitromethane. 18

Figure S14. PXRD patterns: a) calculated pattern from (**tpyram**)(**135tfib**) single crystal data, b) product obtained by grinding **tpyram** and **135tfib** in a 1:1 stoichiometric ratio in the presence of 10.0 μL of nitromethane. 19

Figure S15. PXRD patterns: a) calculated pattern from (**tpyraem**)(**14tfib**) single crystal data, b) product obtained by grinding **tpyr**, **14tfib** and **aem** in a 1:1:1 stoichiometric ratio. 19

Figure S16. PXRD patterns: a) calculated pattern from (**tpyraem**)$_2$(**135tfib**)$_5$ single crystal data, b) product obtained by grinding **tpyr**, **135tfib** and **aem** in a 1:2.5:1 stoichiometric ratio at 20 Hz, c) product obtained by grinding **tpyr**, **135tfib** and **aem** in a 1:2.5:1 stoichiometric ratio at 15 Hz, d) product obtained by cocrystallizing **tpyraem** and **135tfib** in a 1:1 stoichiometric ratio from chloroform. 20

Figure S17. PXRD patterns: a) calculated pattern from **tpyram** form I single crystal data, b) bulk product obtained by recrystallizing the starting **tpyram** material from ethanol, c) calculated pattern from **tpyram** form II single crystal data, d) starting **tpyram** material (obtained from methanol), e) bulk product obtained by recrystallizing the starting **tpyram** material from chloroform. 20

Figure S18. Comparison of calculated and experimental PXRD patterns of the isomorphous (**tpyram**)$_2$(**14tfib**)$_3$ and (**tpyram**)$_2$(**14tfib**)$_3$ cocrystals. 21

Figure S19. DSC curve of **tpyr**. 22

Figure S20. DSC curve of **tpyram** form I. 22

Figure S21. DSC curve of **tpyram** form II. 23

Figure S22. DSC curve of (**tpyr**)$_2$(**14tfib**). 23

Figure S23. DSC curve of (**tpyr**)(**135tfib**). 24

Figure S24. DSC curve of (**tpyram**)$_2$(**14tfib**)$_3$. 24

Figure S25. DSC curve of (**tpyram**)$_2$(**14tfbb**)$_3$. 25

Figure S26. DSC curve of (**tpyram**)(**135tfib**). 25

Figure S27. DSC curve of (**tpyraem**)(**14tfib**). 26

Figure S28. DSC curve of (**tpyraem**)$_2$(**135tfib**)$_5$. 26

EXPERIMENTAL DETAILS

The **tpyram** Schiff base was synthesized by dissolving 581 mg **tpyr** (5.00 mmol) in 5.0 mL of hot methanol, after which 485 µL of **am** (514 mg, 5.02 mmol) and 20.0 µL of concentrated hydrochloric acid (w = 36 %, 0.23 mmol) were mixed in. The solution was then left to crystallize at room temperature. Repeated experiments have yielded colorless crystalline plates and blocks that were determined to be pure **tpyram** form II (see Figure S9, below)

Since **tpyraem** doesn't crystallize at room temperature and near-atmospheric pressures, a solution of the Schiff base was made by dissolving 581 mg of **tpyr** (5.00 mmol) in 5.0 mL of methanol followed by the addition of 660 µL of 4-(2-aminoethyl)morpholine (5.03 mmol) and of 20.0 µL of concentrated hydrochloric acid (w = 36 %, 0.23 mmol). This solution can be used as-is, as a solution of approximately 1 mmol mL^{-1} **tpyraem**. The residue after solvent evaporation darkens, but can be reused up to approximately a week *via* the addition of appropriate amounts of methanol, ethanol or dichloromethane. After that, however, the same solution leads to clouding and leaves oily brown droplets in the experimentally used solvents: ethanol, chloroform, dichloromethane, or *n*-hexane mixed with diethyl ether.

In this work, the initial methanol solution of **tpyraem** was left to evaporate at room temperature. The remaining liquid substance was then dissolved in 5.0 mL of methanol, resulting in a solution of approximately 1 mmol mL^{-1} **tpyraem**.

SOLUTION SYNTHESES

tpyram form I

20.9 mg of **tpyram** (0.104 mmol) was dissolved in 2.0 mL of hot acetonitrile, after which 13.0 µL of **ipfb** were added. The solution was then left to crystallize at room temperature, yielding crystalline needles that were determined to be pure **tpyram** form I (see Figure S8 below).

Bulk product for DSC experiments was obtained by mixing 15.0 mg of **tpyram** (0.0749 mmol) with 1.0 mL of ethanol and heating until dissolved. The solution was then left to crystallize at room temperature, with crystalline product sampled from the crystallization vessel walls.

(tpyr)$_2$(14tfib)

11.6 mg of **tpyr** (0.100 mmol) and 20.0 mg of **14tfib** (0.0500 mmol) were dissolved in a solvent mixture of 1.0 mL *n*-hexane and 2.0 mL diethyl ether. The solution was then left to crystallize at room temperature.

(tpyr)(135tfib)

20.0 mg of **tpyr** (0.172 mmol) and 43.8 mg of **135tfib** (0.0860 mmol) were dissolved in a solvent mixture of 1.0 mL methanol and 1.0 mL acetonitrile. The solution was then left to crystallize at room temperature.

(tpyram)$_2$(14tfib)$_3$

20.0 mg of **tpyram** (0.0999 mmol) and 37.7 mg of **14tfib** (0.0938 mmol) were dissolved in 1.0 mL of acetonitrile. The solution was then left to crystallize at room temperature.

(tpyram)(135tfib)

31.8 mg of **tpyram** (0.159 mmol) and 81.6 mg of **135tfib** (0.160 mmol) were dissolved in a solvent mixture of 1.5 mL acetonitrile and 1.5 mL methanol. The mixture was heated until the solid reactants fully dissolved, and then left to crystallize at room temperature.

(tpyram)$_2$(14tfbb)$_3$

20.0 mg of **tpyram** (0.0999 mmol) and 28.9 mg of **14tfbb** (0.0938 mmol) were dissolved in 2.0 mL of ethanol. The solution was then left to crystallize at room temperature.

(tpyraem)(14tfib)

42.2 mg of **14tfib** (0.105 mmol) was dissolved in a solvent mixture of 1.0 mL *n*-hexane and 1.0 mL diethyl ether, after which 100 μL of the **tpyr4aem** solution was mixed in. The solution was then left to crystallize at room temperature.

(tpyraem)$_2$(135tfib)$_5$

51.4 mg of **135tfib** (0.101 mmol) was dissolved in 2.0 mL of ethanol, after which 125 μL of the **tpyraem** solution was mixed in. The solution was then left to crystallize at room temperature.

MECHANOCHEMICAL SYNTHESES

Reaction mixtures were placed in a 5 mL stainless steel jar along with optionally a small amount of nitromethane (10.0 µL), and one stainless steel ball 7 mm in diameter. The reaction mixtures were then milled for 20 minutes in a Retsch MM200 Shaker Mill operating at 25 Hz. Experimental details were as follows follow in Table S1:

Table S1. Mechanochemical synthesis parameters and results. The samples were milled for 20 minutes on a Retsch MM200 shaker mill set at 25 Hz vibration frequency, using Retsch 5 mL stainless steel jars and 1 stainless steel ball 7mm in diameter. **tpyraem** was used as a methanol solution, $c = 1$ mmol mL^{-1}.

R1	R2	*m* or *V* (R1)	*m* or *V* (R2)	liquid	*V* (liquid) / µL	result
**tpyr**	**14tfib**	23.2 mg	40.0 mg	CH_3NO_2	10.0	**(tpyr)**$_2$**(14tfib)**
**tpyr**	**135tfib**	10.0 mg	43.9 mg	CH_3NO_2	10.0	**(tpyr)(135tfib)**
**tpyram**	**14tfib**	20.0 mg	60.2 mg	CH_3NO_2	10.0	**(tpyram)**$_2$**(14tfib)**$_3$
**tpyram**	**14tfbb**	20.0 mg	46.1 mg	CH_3NO_2	10.0	**(tpyram)**$_2$**(14tfbb)**$_3$
**tpyram**	**135tfib**	20.0 mg	50.9 mg	CH_3NO_2	10.0	**(tpyram)(135tfib)**
**tpyram**	**13tfib**	20.0 mg	14.5 µL	CH_3NO_2	10.0	liquid
**tpyram**	**12tfib**	20.0 mg	37.7 mg	CH_3NO_2	10.0	liquid
**tpyram**	**ipfb**	20.0 mg	13.5 µL	CH_3NO_2	10.0	liquid
**tpyraem**	**14tfib**	100.0 µL	40.2 mg			liquid

Because cocrystals of **tpyraem** and **14tfib** or **135tfib** were not successfully obtained by these experiments, we obtained them by instead milling all the precursor reactants in one-pot reactions as follows:

(tpyraem)(14tfib)

40.2 mg of **14tfib** (0.100 mmol) and 11.6 mg of **tpyr** (0.100 mmol) were placed in a Retsch 5 mL stainless steel jar along with 1 stainless steel ball 7 mm in diameter. 13.5 µL of **aem** (0.103 mmol) was added to the mixture, and it was then milled for 20 minutes on a Retsch MM200 shaker mill set to 25 Hz vibration frequency.

(tpyraem)$_2$(135tfib)$_5$

127.2 mg of **135tfib** (0.250 mmol) and 11.6 mg of **tpyr** (0.100 mmol) were placed in an FTS 15 mL stainless steel jar along with 2 stainless steel balls 7 mm in diameter. 13.5 µL of **aem** (0.103 mmol) was added to the mixture, and it was then milled for 20 minutes on a Retsch MM200 shaker mill set to 20 Hz vibration frequency.

THERMAL ANALYSIS

DSC analysis was performed on a Mettler-Toledo DSC823 module. The samples were placed in sealed aluminium pans (40 μL) with one pinhole made in the top cover. The samples were heated in flowing nitrogen from 25 °C to 300 °C (DSC 823) at a heating rate of 10 °C min^{-1}. Data analysis was performed using the program package Mettler $STAR^{e}$ Software 15.00.[1]

POWDER X-RAY DIFFRACTION EXPERIMENTS

PXRD experiments were performed on a PHILIPS PW 1840 X-ray diffractometer or a Malvern PANalytical Aeris X-ray diffractometer. Data collection the PHILIPS diffractometer was performed using the program package Philips X'Pert.[2] Both diffractometers were equipped with copper anode X-ray tubes, using Cu$K\alpha$1 (1.54056 Å) radiation at 40 kV and 40 mA (PHILIPS) or at 40 kV and 15 mA (Aeris). The angular range was from 3 to 40° (2θ) with a step size of 0.03° (PHILIPS), or from 5 to 40° (2θ) with an interpolated step size of 0.00543322° (Aeris). Data analysis was performed using the program package Data Viewer.[3]

SINGLE-CRYSTAL X-RAY DIFFRACTION EXPERIMENTS

The crystal and molecular structures of the prepared samples were determined by single crystal X-ray diffraction. Details of data collection and crystal structure refinement are listed in Table S2. Diffraction measurements were made on a Rigaku Synergy XtaLAB X-ray diffractometer with graphite-monochromated MoKα (λ = 0.71073Å) radiation. The data sets were collected using the ω scan mode over the 2θ range up to 64°. CrysAlisPro waw employed for data collection, cell refinement, and data reduction.[4] The **tpyram** form II crystal was twinned and was treated as a two-component twin during data reduction and further structure solving. The structures were solved by either SHELXT[5] or SHELXS[6] programs and refined using the SHELXL program.[6] The structural refinement was performed on F^2 using all data. Hydrogen atoms were placed in calculated positions and treated as riding on their parent atoms. All calculations were performed using the WINGX crystallographic suite of programs.[7] The molecular structures of compounds and their molecular packing projections were prepared by Mercury.[8]

References

1. STARe Evaluation Software Version 15.00, Mettler–Toledo GmbH, 2016.
2. Philips X'Pert Data Collector 1.3e, Philips Analytical B. V. Netherlands, 2001; Philips X'Pert Graphic & Identify 1.3e Philips Analytical B. V. Netherlands, 2001; Philips X'Pert Plus 1.0, Philips Analytical B. V. Netherlands, 1999.

3. Data Viewer Version 1.9a, PANalytical B.V. Amelo, The Netherlands, 2018.
4. Rigaku Oxford Diffraction, Gemini CCD system , CrysAlis Pro software, Version 171.41.93a, 2020.
5. G. M. Sheldrick, *Acta Cryst. A*, 2015, **71**, 3–8.
6. (a) G. M. Sheldrick, *Acta Cryst. A*, 2008, **64**, 112–122; (b) G. M. Sheldrick, *Acta Cryst. C*, 2015, **71**, 3–8.
7. L. J. Farrugia, *J. Appl. Cryst.*, 2012, **45**, 849–854.
8. C. F. Macrae, I. J. Bruno, J. A. Chisholm, P. R. Edgington, P. McCabe, E. Pidcock, L. Rodriguez-Monge, R. Taylor, J. v. d. Streek and P. A. Wood, *J. Appl. Crystallogr.* **2008**, 41, 466.

Table S2. Crystal data and refinement details for the prepared compounds.

	(tpyr)$_2$(14tfib)	(tpyr)(135tfib)	(tpyram)$_2$(14tfib)$_3$
Molecular formula	$(C_5H_8OS)_2(C_6F_4I_2)$	$(C_5H_8OS)(C_6F_3I_3)$	$(C_9H_{16}N_2OS)_2(C_6F_4I_2)_3$
M_r	634.21	625.93	1606.17
Crystal system	monoclinic	triclinic	monoclinic
Space group	$P2_1/c$	$P\bar{1}$	$C2/c$
Crystal data:			
a / Å	11.8336(9)	7.1554(3)	27.9796(12)
b / Å	6.9449(3)	9.3062(4)	5.9820(2)
c / Å	13.7679(9)	12.3820(4)	30.7717(12)
α / °	90	78.611(3)	90
β / °	113.540(8)	87.745(3)	113.341(5)
γ / °	90	89.626(3)	90
V / Å^3	1037.33(13)	807.65(6)	4728.9(4)
Z	2	2	4
D_{calc} / g cm^{-3}	2.030	2.574	2.256
$\lambda(MoK_\alpha)$ / Å	0.71073	0.71073	0.71073
T / K	295	295	295
Crystal size / mm^3	0.54 x 0.21 x 0.19	0.61 x 0.58 x 0.14	0.34 x 0.21 x 0.14
μ / mm^{-1}	3.276	5.953	4.114
$F(000)$	604	568	3000
Refl. collected/unique	11942 / 3461	9516 / 2825	16735 / 4142
Parameters/restraints	119 / 0	172 / 0	326 / 0
$\Delta\rho_{max}$, $\Delta\rho_{min}$ / e Å^{-3}	0.702; –2.383	1.510; –1.906	0.624; –0.795
$R[F^2 > 4\sigma(F^2)]$	0.0589	0.0731	0.0260
w$R(F^2)$	0.1675	0.2492	0.0609
Goodness-of-fit, S	1.011	1.153	1.046

Table S2. continued

	(tpyram)$_2$(14tfbb)$_3$	(tpyram)(135tfib)	(tpyraem)(14tfib)
Molecular formula	$(C_9H_{16}N_2OS)_2(C_6F_4Br_2)_3$	$(C_9H_{16}N_2OS)(C_6F_3I_3)$	$(C_{11}H_{20}N_2OS)(C_6F_4I_2)$
M_r	1324.23	710.06	630.21
Crystal system	monoclinic	monoclinic	triclinic
Space group	$C2/c$	Cc	$P\bar{1}$
Crystal data:			
a / Å	27.0478(18)	26.9360(6)	6.1492(2)
b / Å	5.8412(3)	9.2880(1)	10.4539(3)
c / Å	30.896(2)	34.3378(10)	17.3615(5)
α / °	90	90	79.006(3)
β / °	114.030(8)	102.745(2)	88.619(2)
γ / °	90	90	77.648(3)
V / Å^3	4458.3(6)	8379.0(3)	1070.05(6)
Z	4	16	2
D_{calc} / g cm^{-3}	1.973	2.251	1.956
$\lambda(MoK_\alpha)$ / Å	0.71073	0.71073	0.71073
T / K	295	295	295
Crystal size / mm^3	0.47 x 0.31 x 0.18	0.61 x 0.42 x 0.29	0.56 x 0.49 x 0.20
μ / mm^{-1}	5.586	4.608	3.080
$F(000)$	2568	5280	604
Refl. collected/unique	29887 / 7522	99501 / 28466	22804 / 7235
Parameters/restraints	281 / 0	901 / 2	244 / 0
$\Delta\rho_{max}$, $\Delta\rho_{min}$ / e Å^{-3}	0.878; –0.823	1.022; –1.404	1.003; –0.898
$R[F^2 > 4\sigma(F^2)]$	0.0704	0.0424	0.0499
w$R(F^2)$	0.1216	0.1111	0.1369
Goodness-of-fit, S	0.991	1.069	1.038

Table S2. continued

	(**tpyraem**)$_2$(**135tfib**)$_5$	**tpyram** form I	**tpyram** form II
Molecular formula	$(C_{11}H_{20}N_2OS)_2(C_6F_3I_3)_5$	$C_9H_{16}N_2OS$	$C_9H_{16}N_2OS$
M_r	3005.50	200.30	200.30
Crystal system	monoclinic	orthorhombic	monoclinic
Space group	$C2/c$	$P2_12_12_1$	$P2_1/n$
Crystal data:			
a / Å	34.3449(13)	6.2805(4)	12.0117(19)
b / Å	9.3018(2)	10.3321(5)	7.8750(11)
c / Å	24.4064(8)	16.3478(8)	12.395(2)
α / °	90	90	90
β / °	105.424(4)	90	117.18(2)
γ / °	90	90	90
V / Å^3	7516.3(4)	1060.82(10)	1043.1(3)
Z	4	4	4
D_{calc} / g cm^{-3}	2.656	1.254	1.275
$\lambda(MoK_\alpha)$ / Å	0.71073	0.71073	0.71073
T / K	295	295	295
Crystal size / mm^3	0.59 x 0.26 x 0.20	0.56 x 0.28 x 0.20	0.75 x 0.57 x 0.18
μ / mm^{-1}	6.309	0.270	0.275
$F(000)$	5432	432	432
Refl. collected/unique	40591 / 12553	11101 / 3070	2727 / 2727
Parameters/restraints	409 / 0	118 / 0	119
$\Delta\rho_{max}$, $\Delta\rho_{min}$ / e Å^{-3}	1.602; –1.411	0.656; –0.201	0.276; –0.266
$R[F^2 > 4\sigma(F^2)]$	0.0476	0.0797	0.0550
w$R(F^2)$	0.1312	0.2319	0.1566
Goodness-of-fit, S	1.098	1.126	0.984

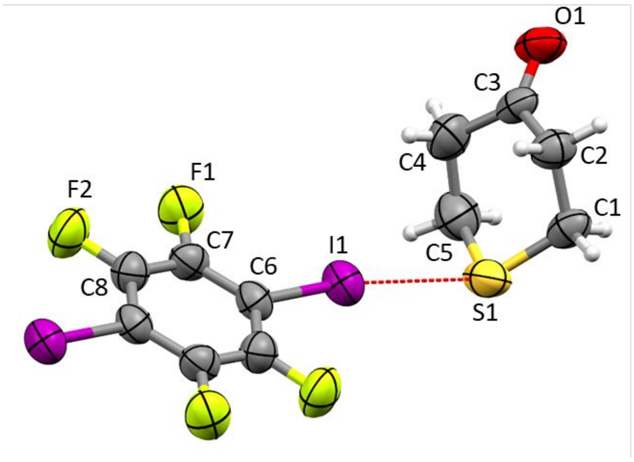

Figure S1. Molecular structure of (**tpyr**)$_2$(**14tfib**) showing the atom-labeling scheme. Displacement ellipsoids are drawn at the 50 % probability level, and H atoms are shown as small spheres of arbitrary radius.

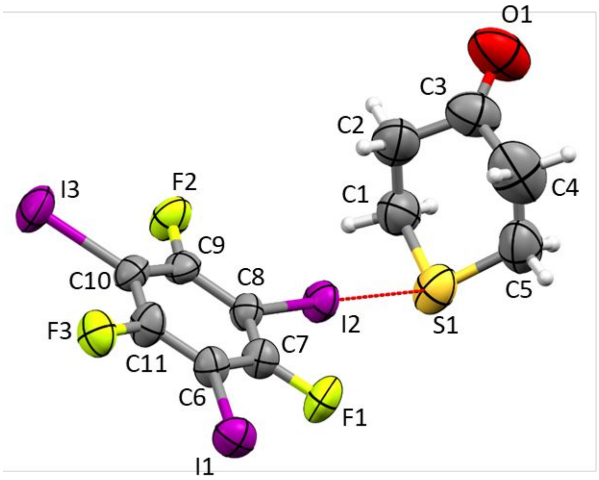

Figure S2. Molecular structure of (**tpyr**)(**135tfib**) showing the atom-labeling scheme. Displacement ellipsoids are drawn at the 50 % probability level, and H atoms are shown as small spheres of arbitrary radius.

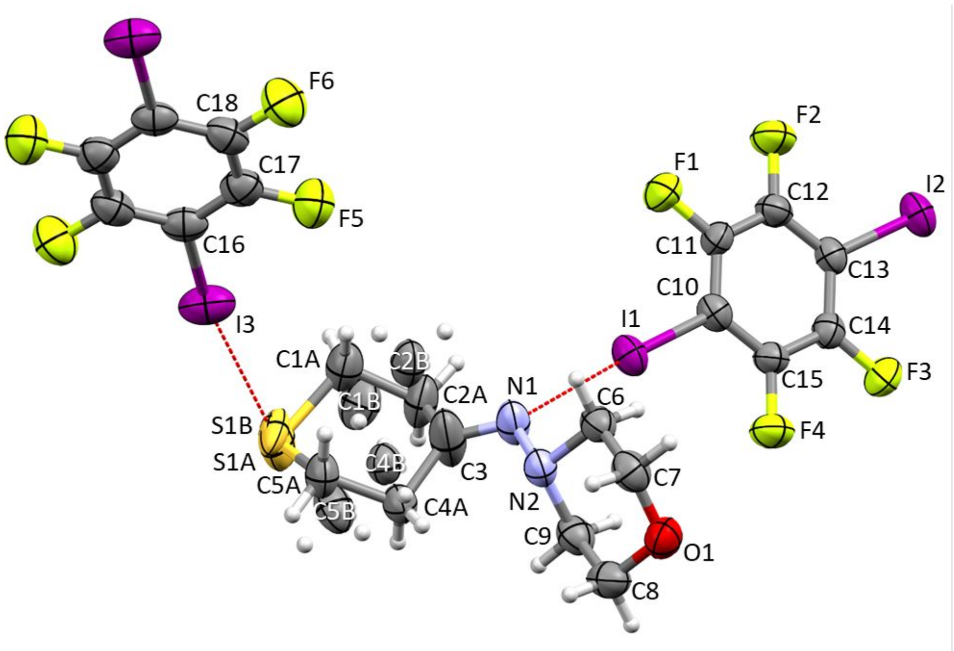

Figure S3. Molecular structure of (**tpyram**)$_2$(**14tfib**)$_3$ showing the atom-labeling scheme. Displacement ellipsoids are drawn at the 50 % probability level, and H atoms are shown as small spheres of arbitrary radius.

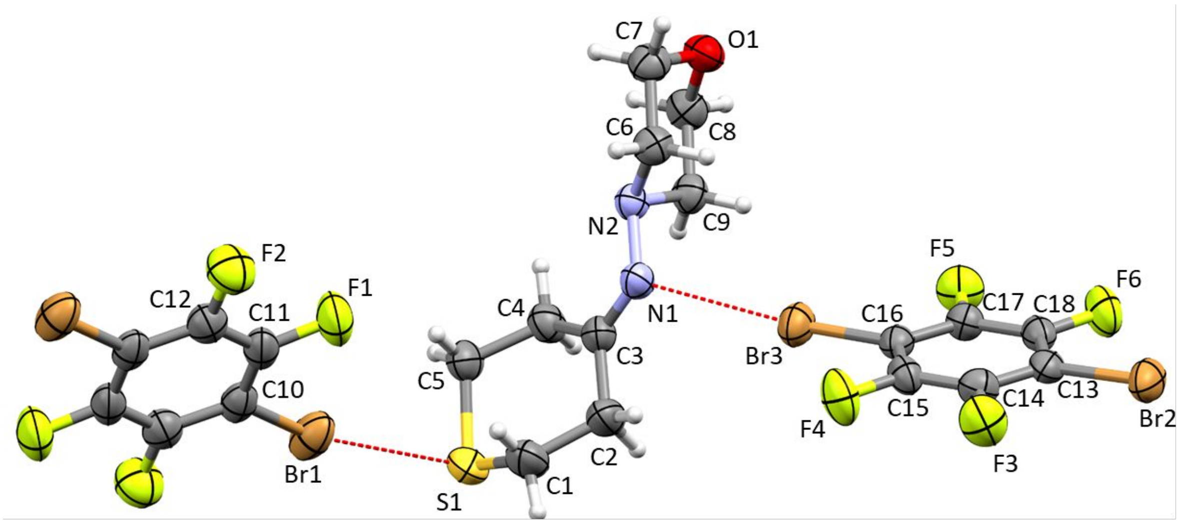

Figure S4. Molecular structure of (**tpyram**)$_2$(**14tfbb**)$_3$ showing the atom-labeling scheme. Displacement ellipsoids are drawn at the 50 % probability level, and H atoms are shown as small spheres of arbitrary radius.

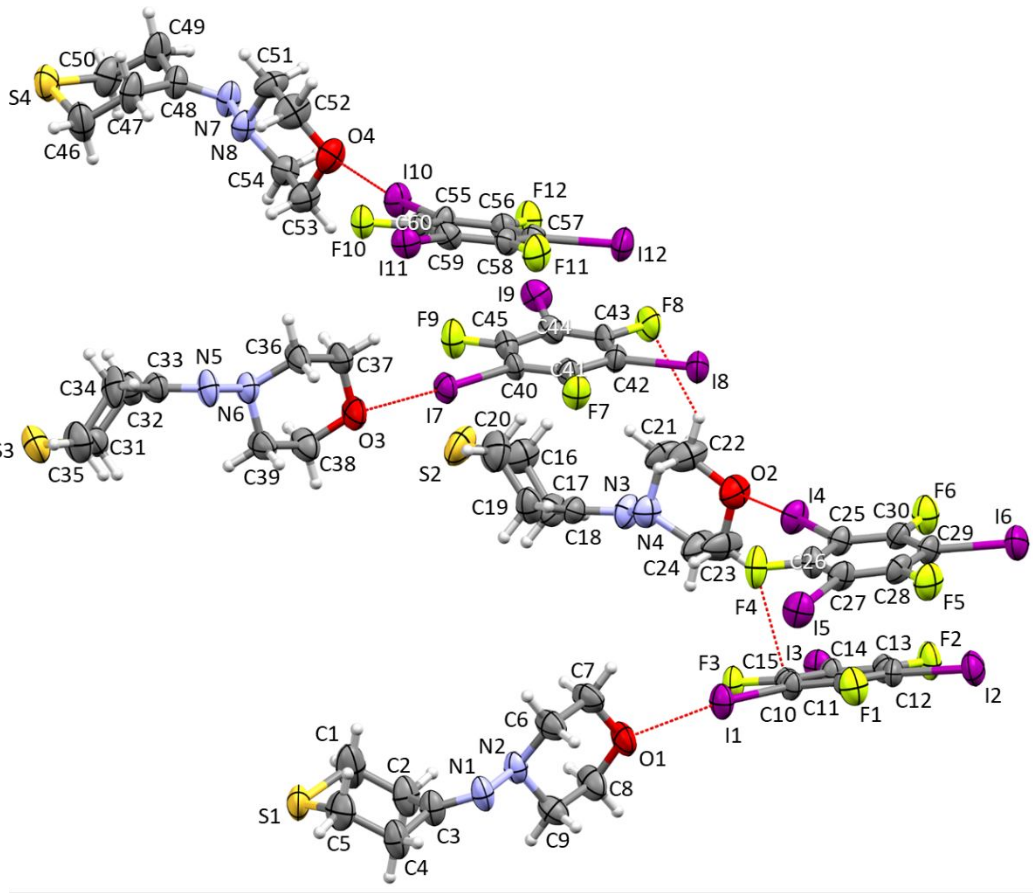

Figure S5. Molecular structure of (**tpyram**)(**135tfib**) showing the atom-labeling scheme. Displacement ellipsoids are drawn at the 50 % probability level, and H atoms are shown as small spheres of arbitrary radius.

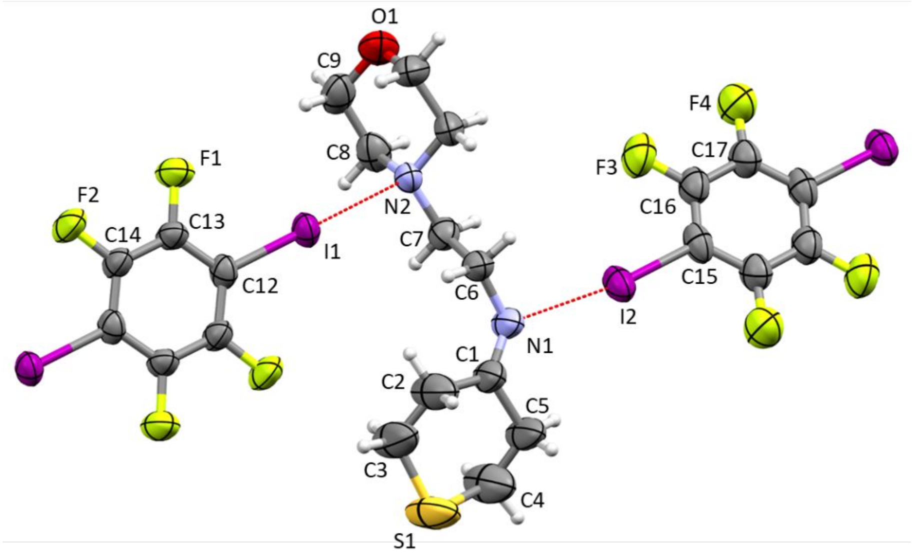

Figure S6. Molecular structure of (**tpyraem**)(**14tfib**) showing the atom-labeling scheme. Displacement ellipsoids are drawn at the 50 % probability level, and H atoms are shown as small spheres of arbitrary radius.

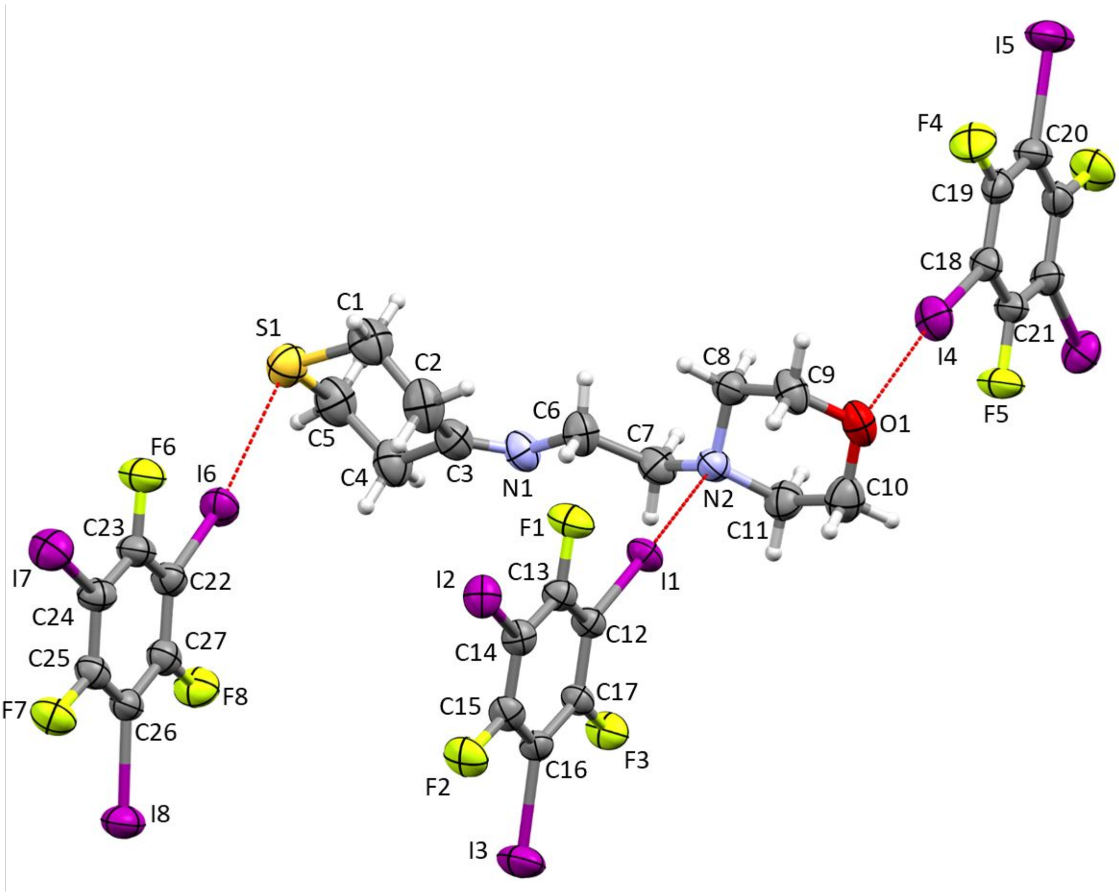

Figure S7. Molecular structure of (**tpyraem**)$_2$(**135tfib**)$_5$ showing the atom-labeling scheme. Displacement ellipsoids are drawn at the 50 % probability level, and H atoms are shown as small spheres of arbitrary radius.

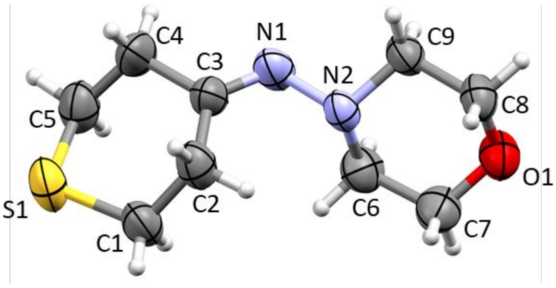

Figure S8. Molecular structure of **tpyram** form I, showing the atom-labeling scheme. Displacement ellipsoids are drawn at the 50 % probability level, and H atoms are shown as small spheres of arbitrary radius.

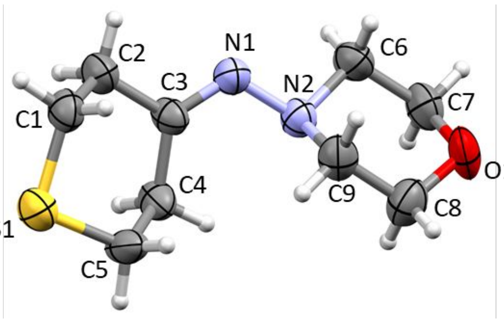

Figure S9. Molecular structure of **tpyram** form II, showing the atom-labeling scheme. Displacement ellipsoids are drawn at the 50 % probability level, and H atoms are shown as small spheres of arbitrary radius.

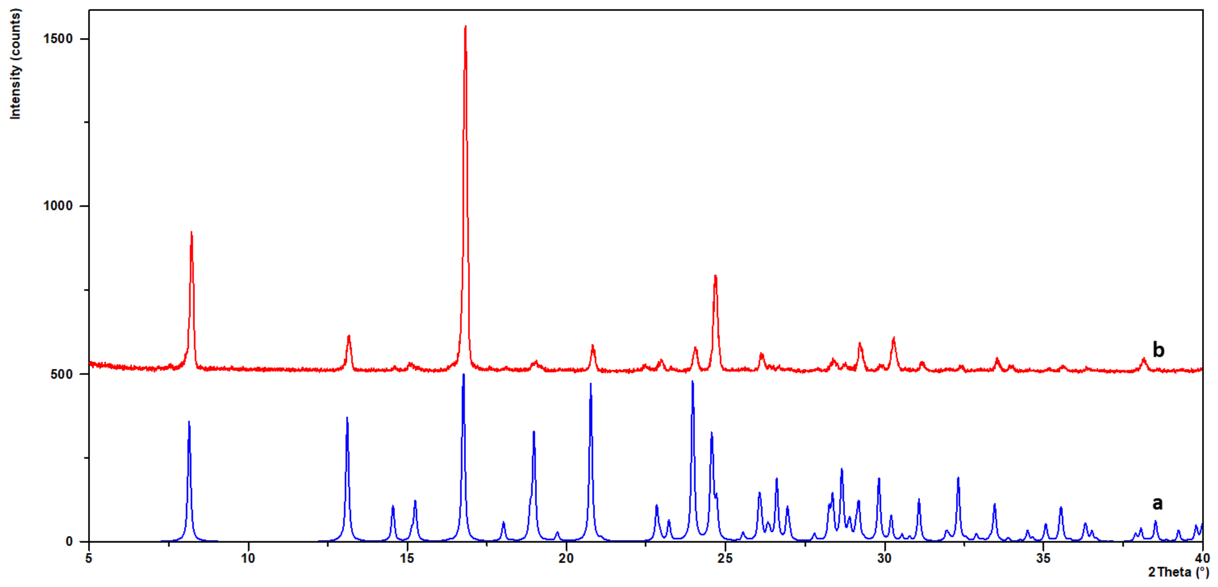

Figure S10. PXRD patterns: a) calculated pattern from (**tpyr**)$_2$(**14tfib**) single crystal data, b) bulk product obtained by grinding **tpyr** and **14tfib** in a 2:1 stoichiometric ratio in the presence of 10.0 μL of nitromethane.

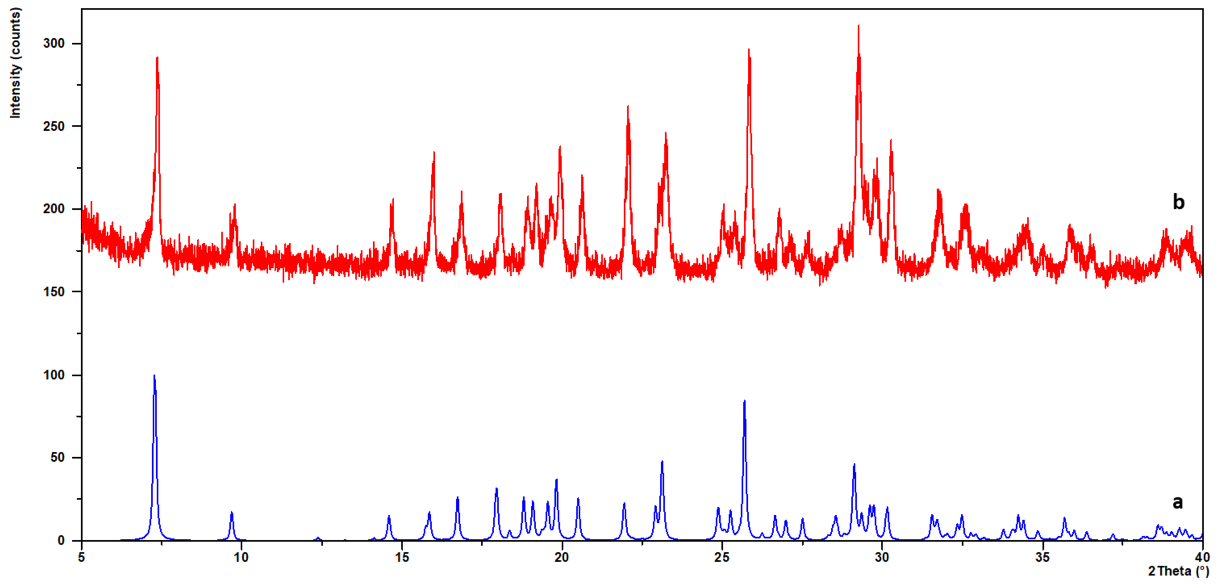

Figure S11. PXRD patterns: a) calculated pattern from (**tpyr**)(**135tfib**) single crystal data, b) bulk product obtained by grinding **tpyr** and **135tfib** in a 1:1 stoichiometric ratio in the presence of 10.0 μL of nitromethane.

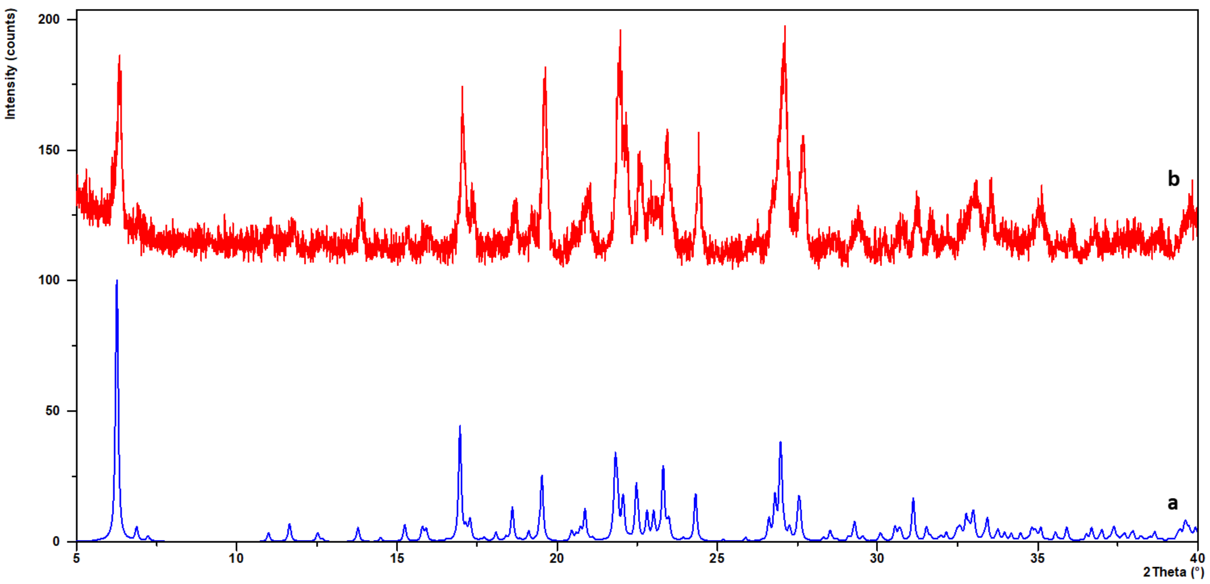

Figure S12. PXRD patterns: a) calculated pattern from (**tpyram**)$_2$(**14tfib**)$_3$ single crystal data, b) product obtained by grinding **tpyram** and **14tfib** in a 2:3 stoichiometric ratio in the presence of 10.0 μL of nitromethane.

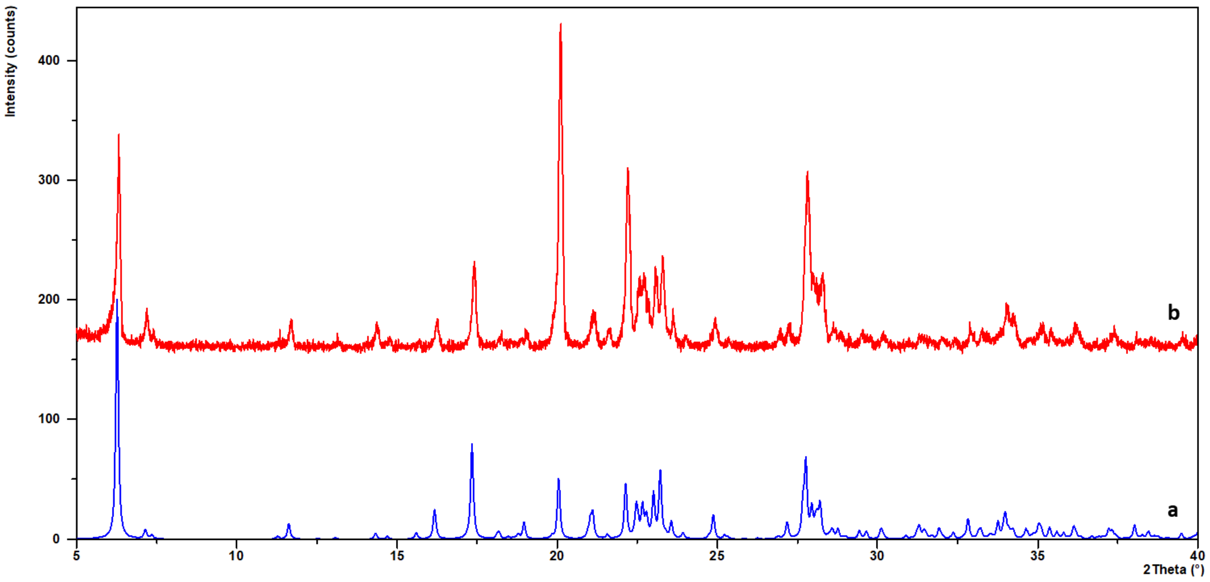

Figure S13. PXRD patterns: a) calculated pattern from (**tpyram**)$_2$(**14tfbb**)$_3$ single crystal data, b) product obtained by grinding **tpyram** and **14tfbb** in a 2:3 stoichiometric ratio in the presence of 10.0 μL of nitromethane.

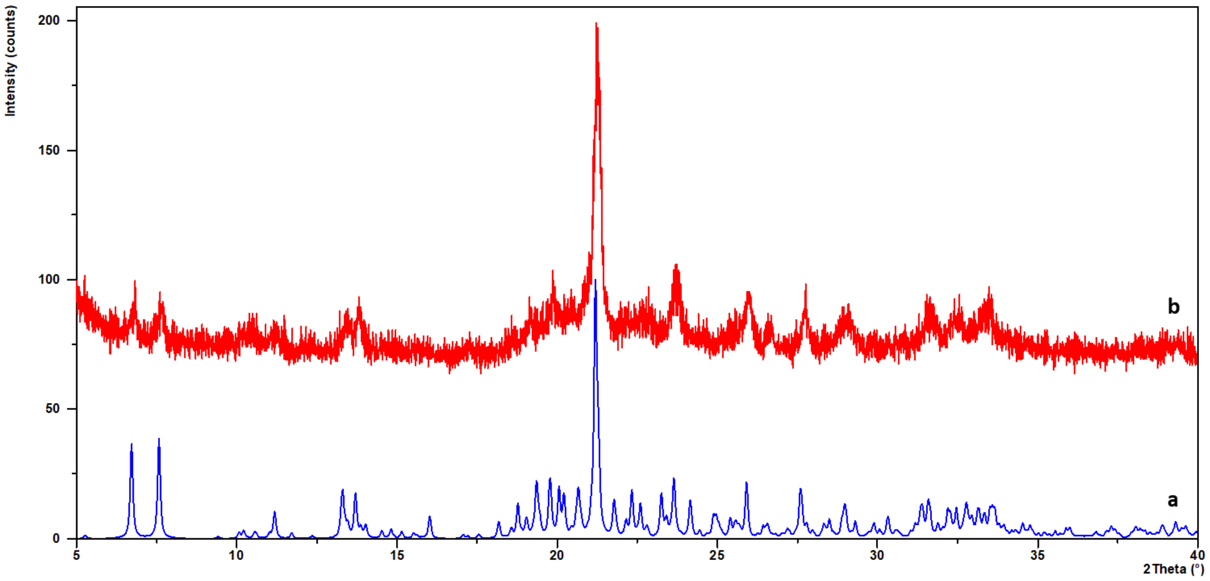

Figure S14. PXRD patterns: a) calculated pattern from (**tpyram**)(**135tfib**) single crystal data, b) product obtained by grinding **tpyram** and **135tfib** in a 1:1 stoichiometric ratio in the presence of 10.0 μL of nitromethane.

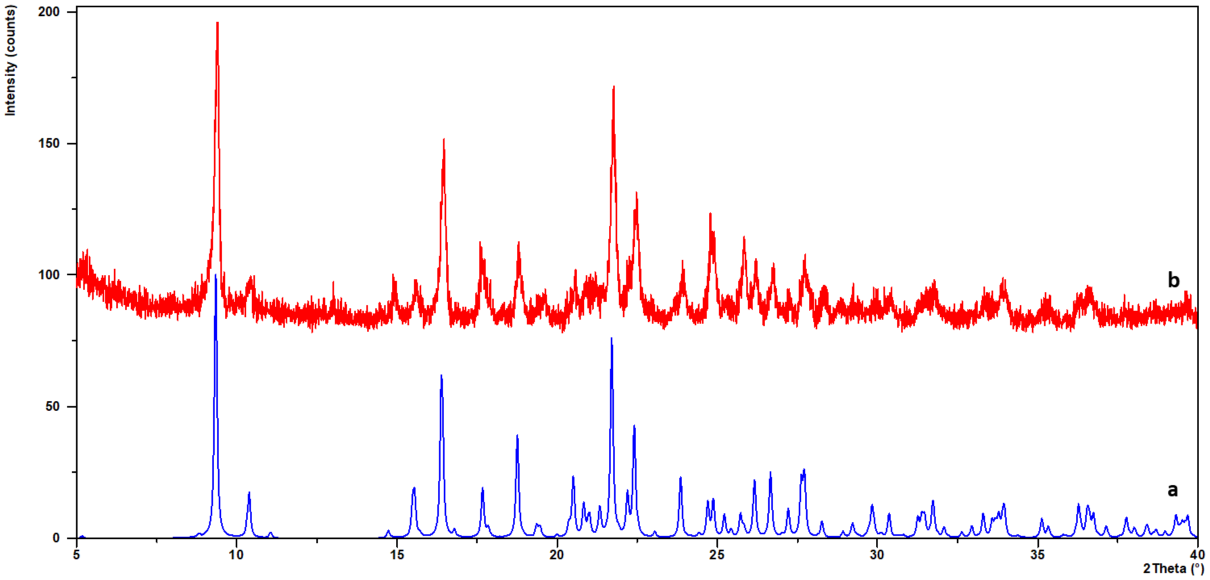

Figure S15. PXRD patterns: a) calculated pattern from (**tpyraem**)(**14tfib**) single crystal data, b) product obtained by grinding **tpyr**, **14tfib** and **aem** in a 1:1:1 stoichiometric ratio.

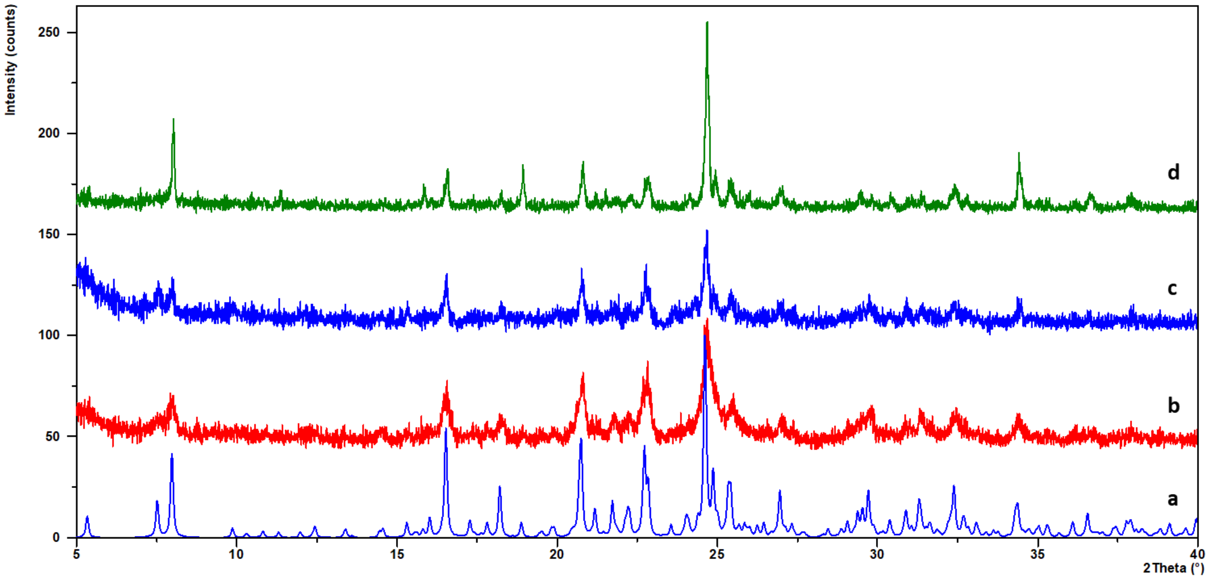

Figure S16. PXRD patterns: a) calculated pattern from (**tpyraem**)$_2$(**135tfib**)$_5$ single crystal data, b) product obtained by grinding **tpyr**, **135tfib** and **aem** in a 1:2.5:1 stoichiometric ratio at 20 Hz, c) product obtained by grinding **tpyr**, **135tfib** and **aem** in a 1:2.5:1 stoichiometric ratio at 15 Hz, d) product obtained by cocrystallizing **tpyraem** and **135tfib** in a 1:1 stoichiometric ratio from chloroform.

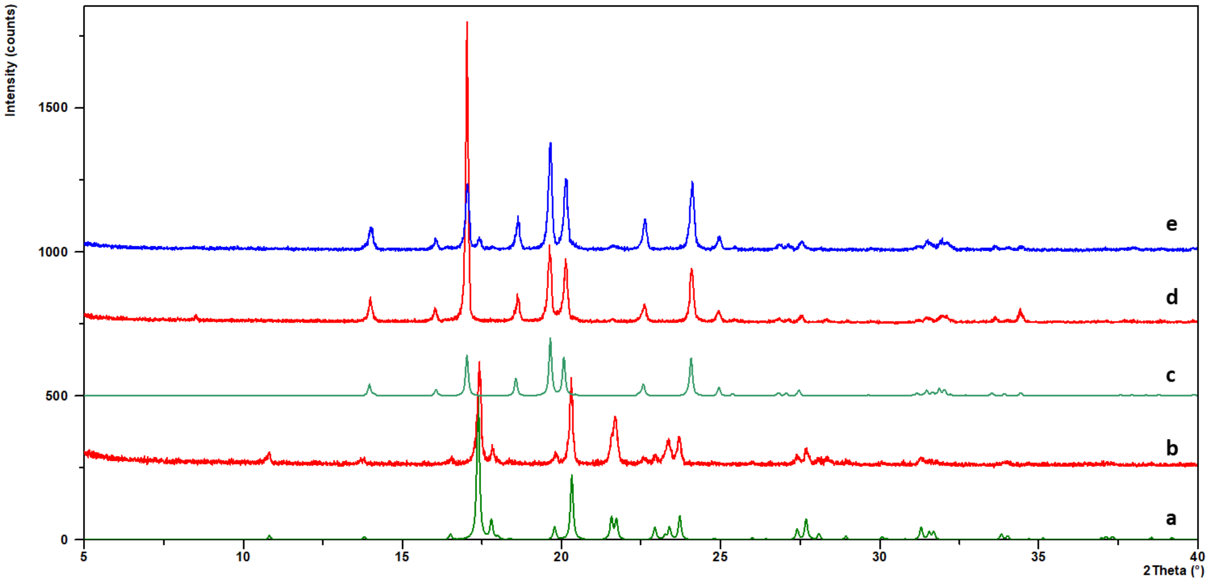

Figure S17. PXRD patterns: a) calculated pattern from **tpyram** form I single crystal data, b) bulk product obtained by recrystallizing the starting **tpyram** material from ethanol, c) calculated pattern from **tpyram** form II single crystal data, d) starting **tpyram** material (obtained from methanol), e) bulk product obtained by recrystallizing the starting **tpyram** material from chloroform.

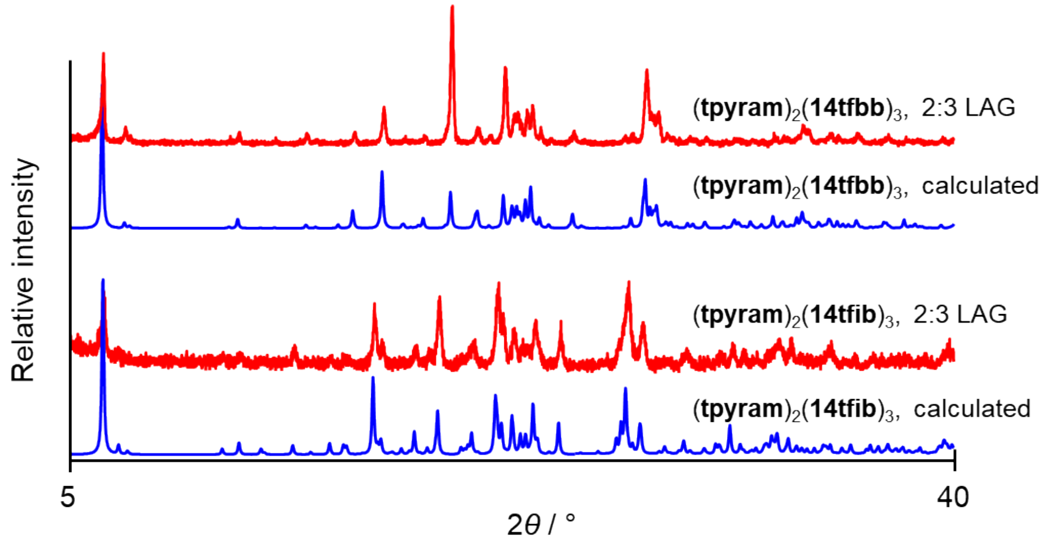

Figure S18. Comparison of calculated and experimental PXRD patterns of the isomorphous **(tpyram)**$_2$(**14tfib**)$_3$ and (**tpyram**)$_2$(**14tfib**)$_3$ cocrystals.

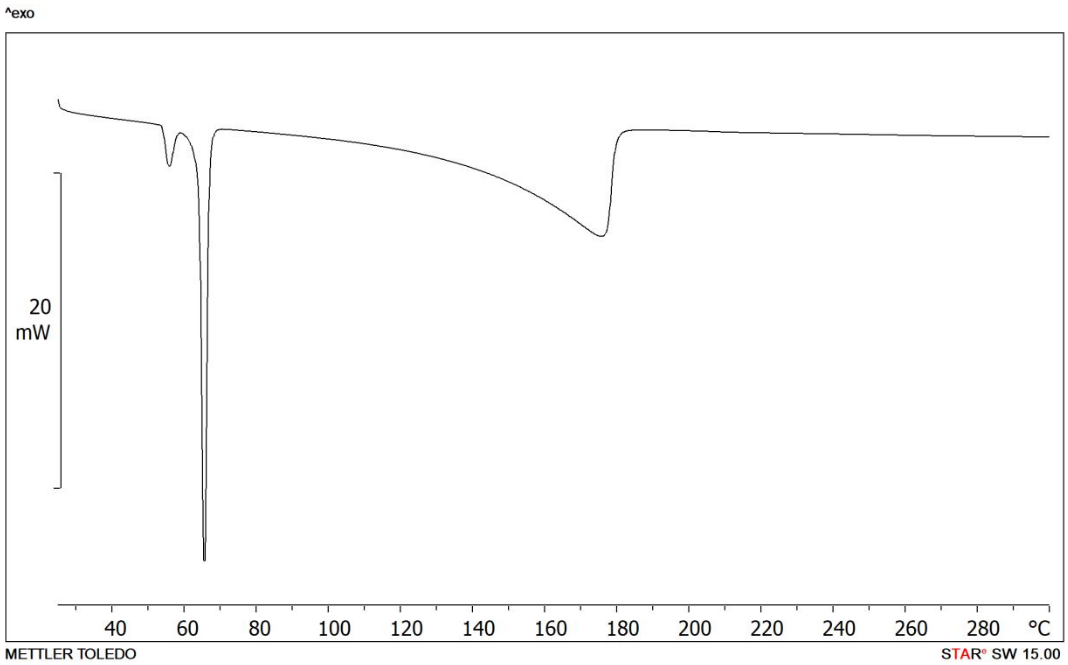

Figure S19. DSC curve of **tpyr**.

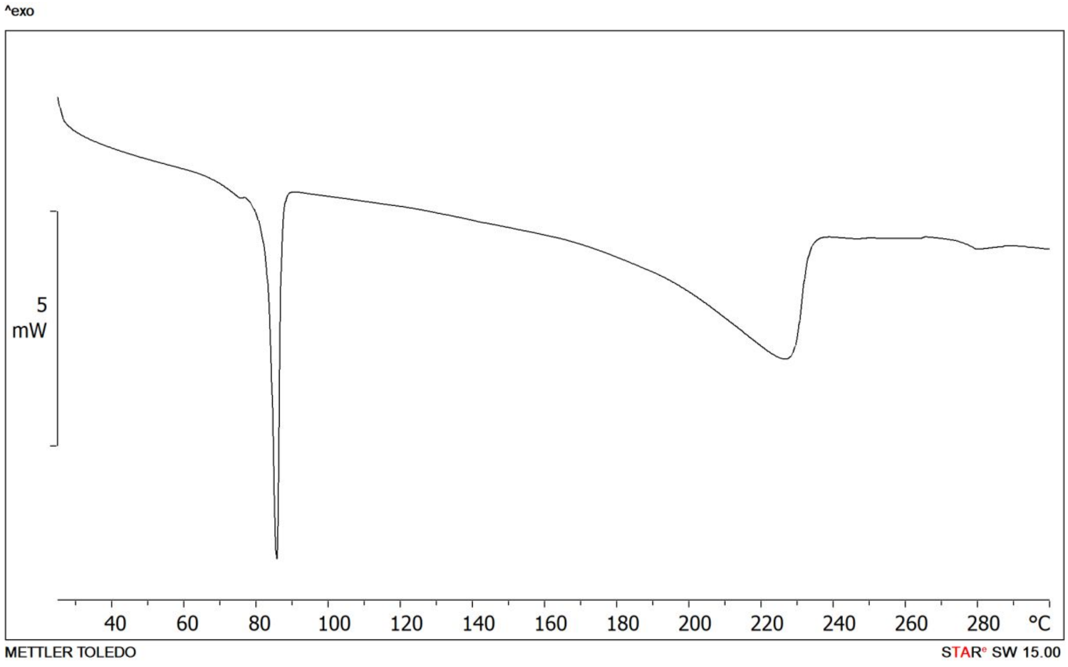

Figure S20. DSC curve of **tpyram** form I.

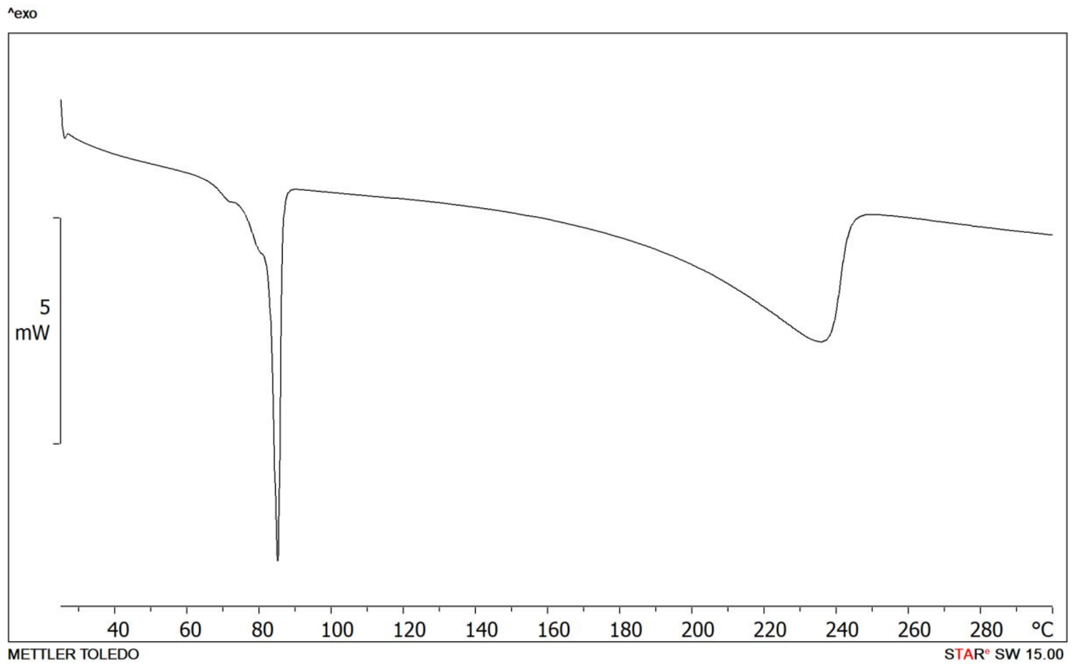

Figure S21. DSC curve of **tpyram** form II.

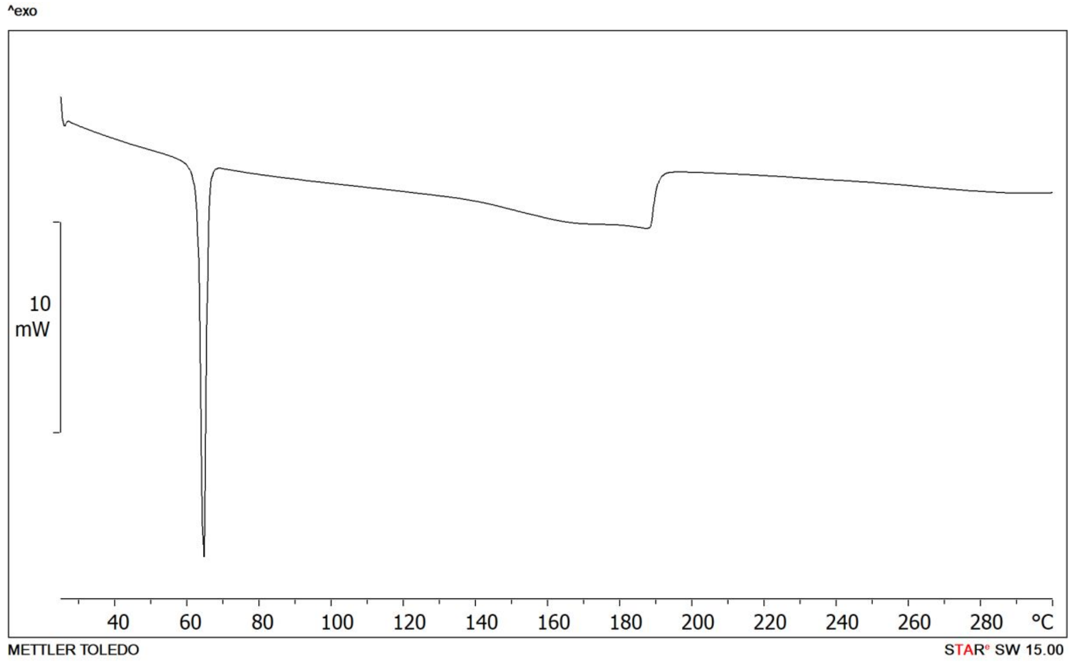

Figure S22. DSC curve of (**tpyr**)$_2$(**14tfib**).

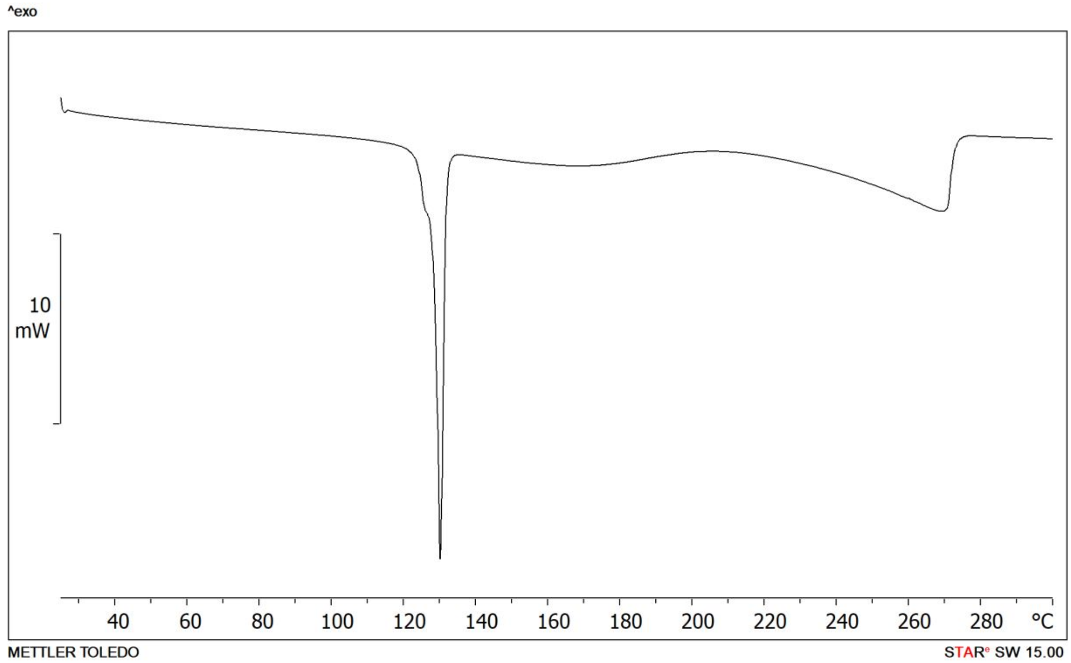

Figure S23. DSC curve of (**tpyr**)(**135tfib**).

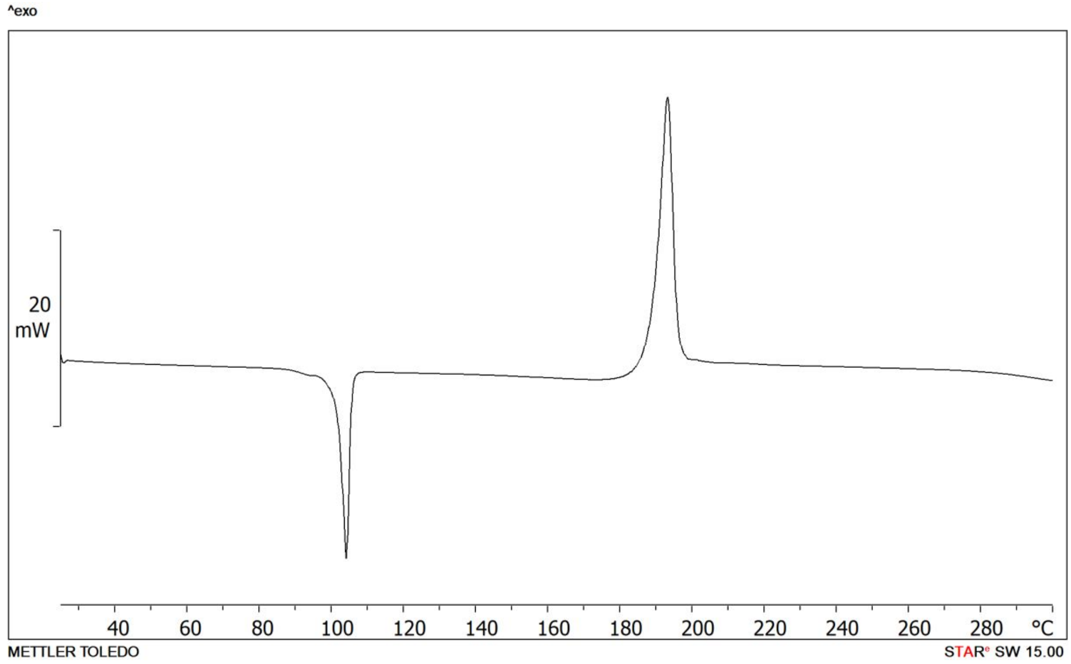

Figure S24. DSC curve of (**tpyram**)$_2$(**14tfib**)$_3$.

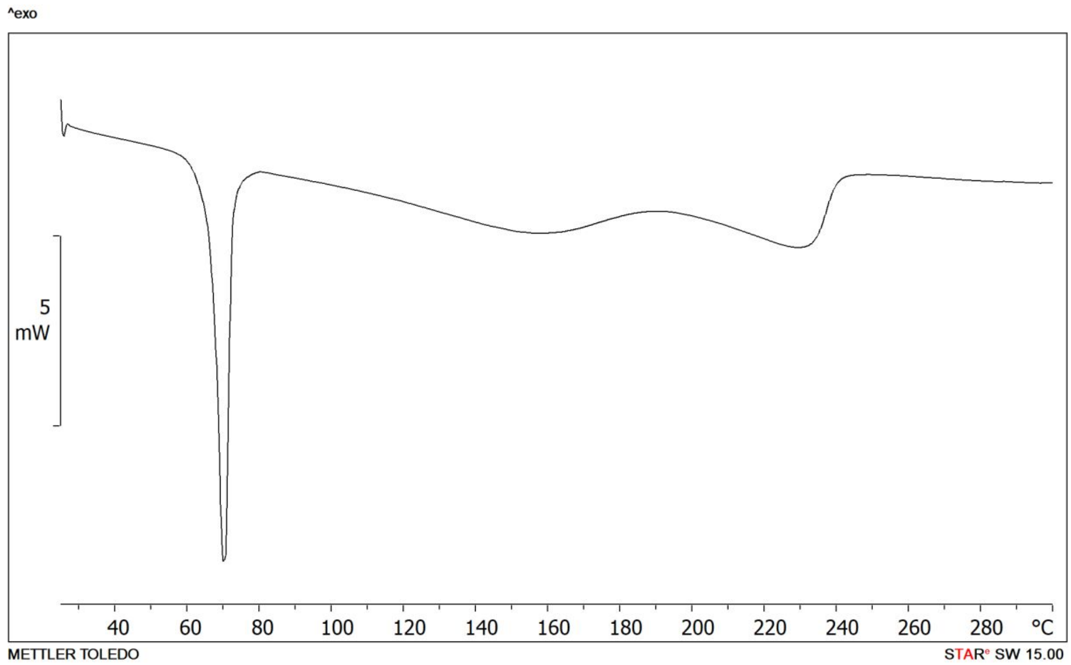

Figure S25. DSC curve of (**tpyram**)$_2$(**14tfbb**)$_3$.

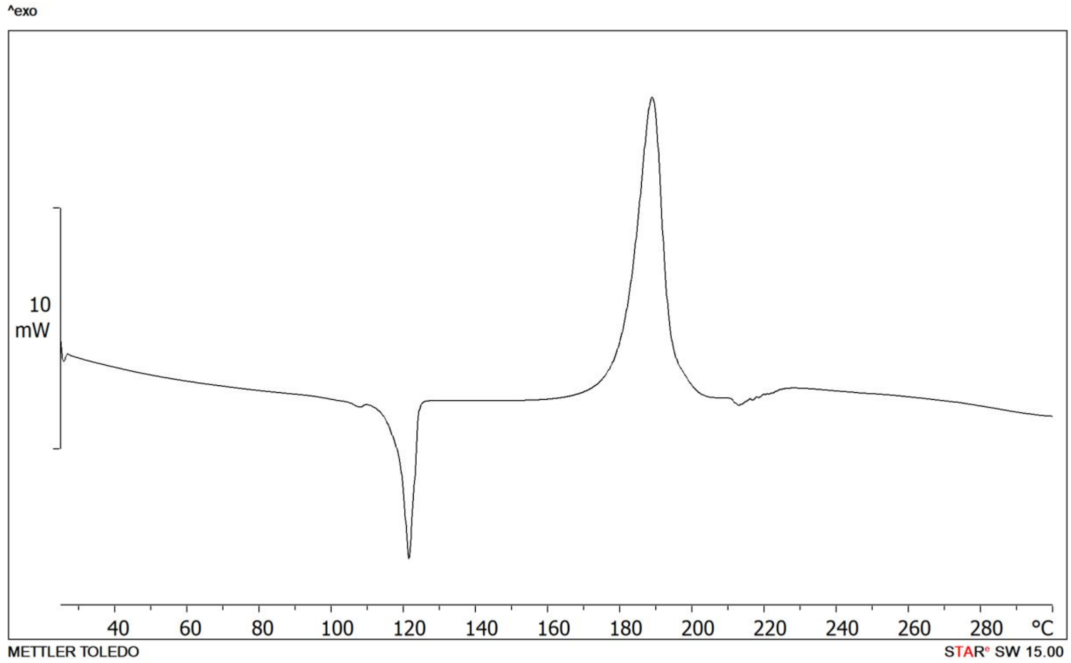

Figure S26. DSC curve of (**tpyram**)(**135tfib**).

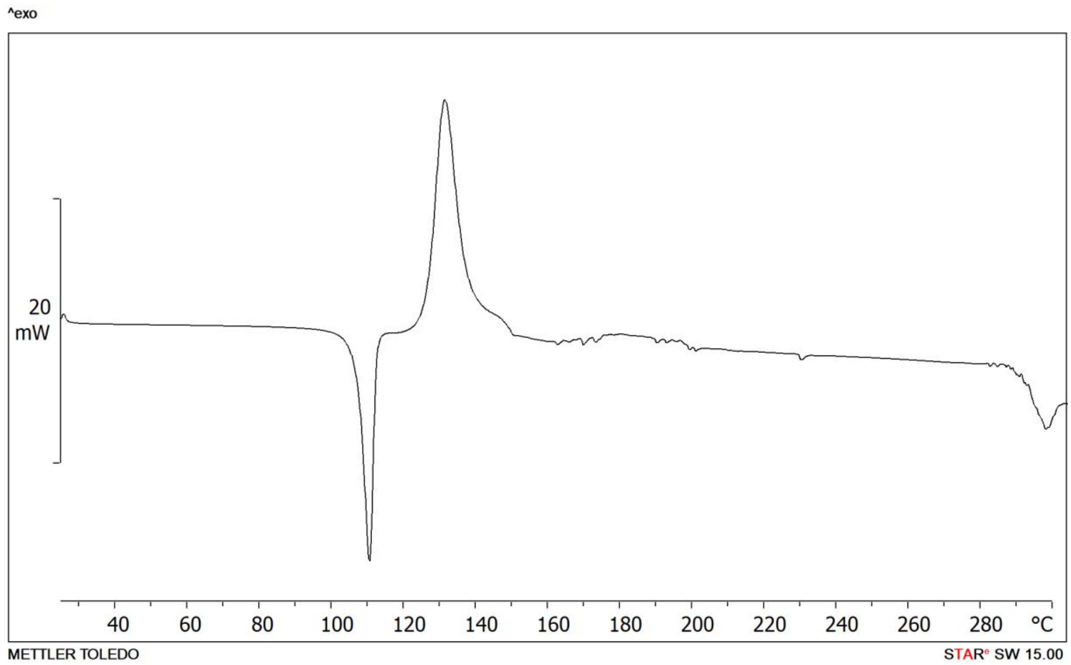

Figure S27. DSC curve of (**tpyraem**)(**14tfib**).

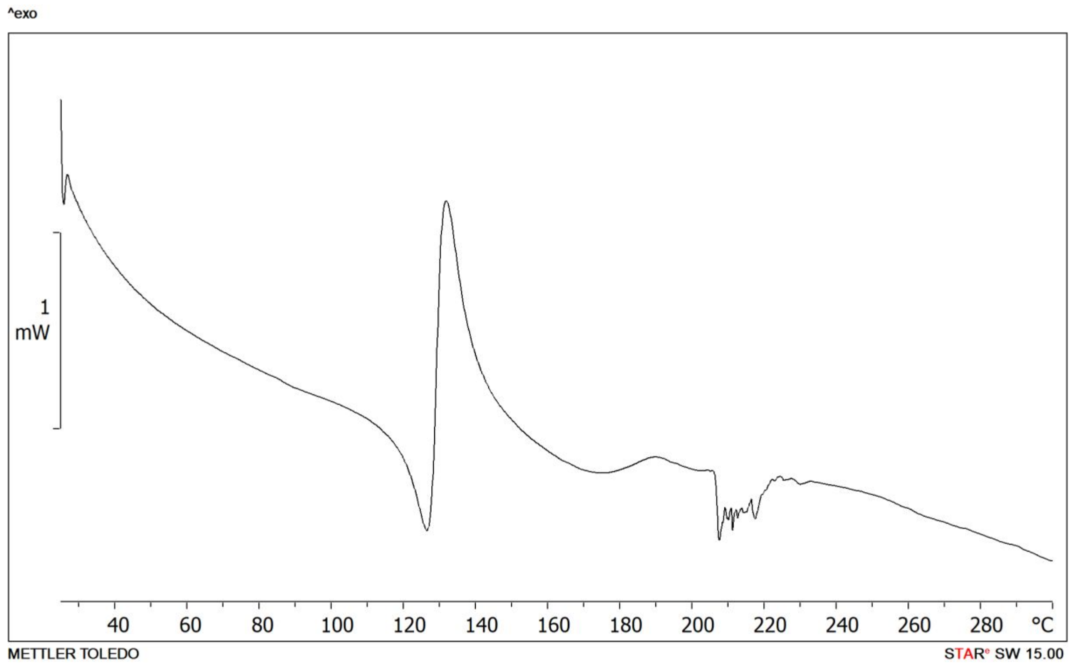

Figure S28. DSC curve of (**tpyraem**)$_2$(**135tfib**)$_5$.
